# Solvent Chemistry in the Electronic Cigarette Reaction Vessel

**DOI:** 10.1038/srep42549

**Published:** 2017-02-14

**Authors:** R. Paul Jensen, Robert M. Strongin, David H. Peyton

**Affiliations:** 1 Department of Chemistry, Portland State University, Portland, Oregon 97207 USA

## Abstract

Knowledge of the mechanism of formation, levels and toxicological profiles of the chemical products in the aerosols (i.e., vapor plus particulate phases) of e-cigarettes is needed in order to better inform basic research as well as the general public, regulators, and industry. To date, studies of e-cigarette emissions have mainly focused on chromatographic techniques for quantifying and comparing the levels of selected e-cigarette aerosol components to those found in traditional cigarettes. E-cigarettes heat and aerosolize the solvents propylene glycol (PG) and glycerol (GLY), thereby affording unique product profiles as compared to traditional cigarettes. The chemical literature strongly suggests that there should be more compounds produced by PG and GLY than have been reported in e-cigarette aerosols to date. Herein we report an extensive investigation of the products derived from vaporizing PG and GLY under mild, single puff conditions. This has led to the discovery of several new compounds produced under vaping conditions. Prior reports on e-cigarette toxin production have emphasized temperature as the primary variable in solvent degradation. In the current study, the molecular pathways leading to enhanced PG/GLY reactivity are described, along with the most impactful chemical conditions promoting byproduct production.

E-cigarettes have emerged as a major public health issue, having been introduced in the U.S. in 2007^1^,^2^,^3^. By 2014, U.S. sales totaled $2.5 billion^4^. While enabling many to stop using traditional cigarettes, a meta-analysis has shown that e-cigarettes are associated with significantly less quitting among smokers than may be commonly believed^5^. Moreover, in a recent longitudinal study, e-cigarettes were consumed by adolescents who would not have otherwise used tobacco products^6^. According to the National Youth Tobacco Survey, in 2013 > 250,000 middle and high school students in the US had used e-cigarettes prior to a traditional cigarette^7^. By 2014, there were >450 brands and >7,770 e-cigarette flavors marketed^8^. In 2015, 16.0% of high school and 5.3% of middle school students reported current use, representing 10- and 5-fold increases since 2011, respectively^9^.

To date, studies of e-cigarette health effects on humans have been limited. For example, studies of the viability of vapers to self-regulate toxin intake by taste alone have typically involved very few human subjects^10^,^11^. Moreover, the long-term effects of vaping cannot be known for at least another decade. There is also a lack of established *in vivo* animal models of e-cigarette exposure^12^.

Knowledge of the mechanisms of formation, levels, and toxicological profiles of the chemical products in the aerosols (i.e., vapor plus particulate phases) from e-cigarettes is needed in order to better inform basic research as well as the general public, regulators, and industry. However, there is currently significant inter-laboratory variation in the published data on the levels of chemical components of electronic cigarette aerosols. As noted by others^11^, this is a reflection of the novelty of the field as well as several major inherent challenges. These include the ongoing emergence of new device configurations, variability in the puffing patterns between individuals, the lack of standardized analytical protocols, and variability between devices.

Studies of e-cigarette emissions have mainly focused on chromatographic techniques for comparing the levels of selected e-cigarette aerosol components^13^. While many studies have reported that e-cigarettes generally have fewer as well as lower levels of the toxins produced from traditional cigarettes, comparing the relative safety of e-cigarettes to traditional cigarettes does not take into account several key factors. For example, e-cigarettes do not combust tobacco, but instead heat and aerosolize nicotine and/or flavorants in the solvents propylene glycol (PG) and glycerol (GLY). Nicotine-free soluble components of e-cigarettes have been linked to dose-dependent loss of lung endothelial barrier function^14^. E-cigarettes therefore produce unique product profiles as compared to traditional cigarettes. Although their study to date in the context of traditional cigarettes is informative, it is also limiting and, moreover, irrelevant to the growing number of young people that are vaping without having ever smoked a cigarette^7^.

The chemistry of PG and GLY has a rich history. The preparation of GLY in 1779 by Scheele, and his determination that it was susceptible to thermal decomposition during simple distillation^15^,^16^,^17^,^18^,^19^, predated even Wöhler’s urea synthesis by half a century. By the mid-19^th^ century, acrolein^20^ and acetic acid^21^ had been identified as products of GLY decomposition. Wurtz synthesized PG in 1859, and determined that it could be oxidized to lactic acid in air in the presence of catalysts^22^. In 1904, Nef provided the foundation for the current understanding of GLY and PG chemistry^23^. He reported that heating GLY afforded hydroxyacetone, acetaldehyde, formaldehyde, acrolein, 3-hydroxypropanal, and a series of acetals. He discovered that GLY formed glycidol upon gentle heating in the presence of acetic acid. Nef also discovered that the decomposition of PG gave propanal. The literature moreover strongly suggests that there should be more compounds produced by PG and GLY than have been investigated in the majority of e-cigarette aerosol studies to date. We propose that since NMR is a technique that can enable relatively broad profiling of compound classes with limited sample perturbation, it will allow the detection and study of overlooked e-cigarette aerosol products as compared to studies that to date have relied nearly exclusively on chromatographic-based methods.

Recently, we reported the discovery of hemiformals **1a** and **1b** (major isomers observed are shown in Fig. 1), products of the reaction of PG/GLY with HCHO that are formed during PG/GLY aerosol generation^24^. Herein, we describe an extensive investigation of the products derived from vaporizing PG and GLY under relatively mild e-cigarette conditions, along with a detailed description of the molecular pathways leading to product formation. Products identified by NMR include glycidol (**2**), an International Agency for Research on Cancer (IARC) Group 2A probable human carcinogen^25^. In addition, we report several products identified for the first time in e-cigarette aerosols such as reactive vinyl alcohol isomers (**3a** and **3b**) and dihydroxyacetone (**4**), the main ingredient in spray tan products that has raised concerns as an inhalation hazard.

## Results and Discussion

### General Methods

#### Aerosol sample production and initial analysis

The e-cigarettes used in this study consisted of two main components, a variable voltage/variable wattage (VV/VW) battery, the Innokin^®^ iTaste VV4, fitted with a KangerTech^®^ Protank-II clearomizer. The clearomizer contained a replaceable bottom heating coil (coils with resistances from 1.8–2.5 Ohms were provided by the manufacturer) embedded in a wick that was covered with the PG/GLY e-liquid during usage. E-liquids were composed of 1:1 v/v PG:GLY solutions, except where indicated. Single puffs of aerosolized e-liquid (50 mL) were drawn via a syringe directly into a DMSO-d_6_ solution for analysis by NMR spectroscopy. Between 6–22 mg of aerosol were collected per puff. Such data from single puff samples was obtained because NMR is non-destructive, meaning that one can signal-average until sufficient signal-to-noise is obtained for the required analyses. Typical parameters included: a 30° observation pulse angle, a 6.2 sec repetition rate, and 64 k data point acquisitions for between 64 and 2048 acquisitions (between 0.12 and 2 hr). Line broadening of 0.3 Hz and a final data size of 64 k real data points were used for data processing. Structure assignments were confirmed by minute addition of authentic standards. This was accomplished by first determining the concentration of the component of interest versus an internal standard, the value of which was used as a benchmark for the added amount of pure standard.

### Results

Figure 2 illustrates that more PG/GLY was consumed as a function of increasing device power. In addition to raising the power settings to directly elevate heating coil temperatures, elongating the puff duration likewise promoted PG/GLY consumption. It is well-known that e-cigarette operating temperatures modulate the extent of PG/GLY degradation^11^. In the context of the investigation herein, the experiments illustrated in Fig. 2 show that the ability of the device to produce aerosol mass and the intensities of novel aerosol product peaks in an NMR spectrum are proportional to the power delivered to the e-cigarette coil.

#### Avoidance of dry coils and burnt e-liquid

Conditions were chosen to avoid drying the heating coil and associated overheating of the e-liquid; it has been proposed that users can detect and self-regulate toxin intake (including HCHO) based on taste^10^. However, it is known that in traditional cigarettes the harsh taste from formaldehyde and other aldehydes is overcome due to the nicotine drive^26^ and to cross-desensitization of transient receptor potential ankyrin subtype 1 (TRPA1) channels in sensory neurons^27^,^28^. Moreover, it has not been shown to what degree the use of flavorants in e-cigarettes dulls or overcomes any harsh taste from toxins.

Nonetheless, several steps were taken to avoid overheating of PG/GLY. First puffs were sampled as single acquisitions, or with 5 min puff intervals or longer between any two puffs, and no primer puffs were used. Extra wicking and cooling of the coil were performed by pulling the syringe (cold puff) without activating the device, again 5 min before the sampling puff. These methods ensured that aerosols were never generated from dried coils, since e-liquid had cooled between puffs and had completely covered the coils prior to drawing any aerosol samples. Also, an average 3–5 s puff duration was used. In addition, the Innokin^®^ iTaste VV4 battery (1000 mAh) that was used herein possesses a variable output of 6.0–15 W and variable voltages of 3.0–6.0 V, and is compatible with a variety of clearomizers. This device is described by the manufacturer as accurate to within 0.1 W with “no fluctuation and unexpected dry hits or burned e-liquid”, and has its highest 15 W setting precisely maintained between 0.8 to 2.5 ohms^29^. Both the Innokin battery and KangerTech^®^ Protank-II clearomizer had received positive reviews from the online vaping community.

#### Identification of aerosol products

**Figure 1:**
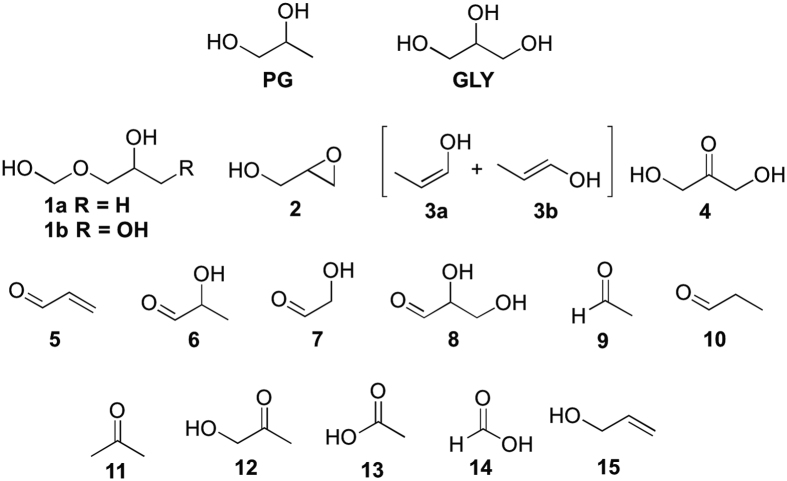
Compounds identified herein by ^1^H NMR in e-cigarette aerosols derived from a single puff from an electronic cigarette. PG = propylene glycol; GLY = glycerol; **1a** = propylene glycol hemiformal (major isomer); **1b** = glycerol hemiformal (major isomer); **2** = glycidol; **3a** = (Z)-prop-1-en-1-ol; **3b** = (E)-prop-1-en-1-ol; **4** = dihydroxyacetone; **5** = acrolein; **6** = lactaldehyde; **7** = glycolaldehyde; **8** = glyceraldehyde; **9** = acetaldehyde; **10** = propanal; **11** = acetone; **12** = hydroxyacetone (acetol); **13** = acetic acid; **14** = formic acid; **15** = allyl alcohol.

The compounds that have been identified in this study are shown in Fig. 1; all of these compounds could be predicted based on the existing PG and GLY literature. Several are reported here for the first time in electronic cigarette aerosols (**3, 4, 6–8**). The determination of others (e.g., **2, 15**) validates their recent discovery in e-cigarette aerosols, and moreover shows that they can form under milder conditions than those reported^25^. Structure assignments for all compounds were validated via spiking with authentic samples when available (Supporting Information).

#### Formaldehyde hemiacetals

Glycerol formal was described by Nef in 1904^23^, and propylene glycol formal was described by Trillat and Cambier in 1894^30^. The production of **1a** and **1b** in e-cigarette aerosols^24^ was thus not surprising, particularly since hemiformals are relatively stable compared to other acyclic hemicacetals. The toxicity of **1a** and **1b** has not been investigated. They reverted to HCHO and PG over a period of hours in aqueous solution. Since **1a** and **1b** have not been separable to date we validated the structure assignment using isotopically labeled CH_3_OCH_2_OH (methoxymethanol) as a model hemiformal. As depicted in Fig. 3, labeled CH_3_OCH_2_OH exhibited analogous peak position as well as the characteristic O-H proton splitting pattern corresponding to those reported for **1a** and **1b**. The ^1^H-^13^C decoupled spectrum B corresponded to that of the product formed by bubbling ^12^CH_2_O in CH_3_OH (spectrum C). Upon spiking with D_2_O (spectrum D) the hydroxyl resonance at 6.14 ppm diminished and the methylene protons at 4.53 ppm appeared as a singlet. The full ^1^H spectrum (E) of CH_3_OCH_2_OH/CH_3_OH in DMSO-d_6_ is shown for completeness. The data are consistent with the predominant NMR peaks as arising from the isomers of **1a** and **1b** shown in Fig. 1.

#### Glycidol (2) and enols (3)

**Figure 2:**
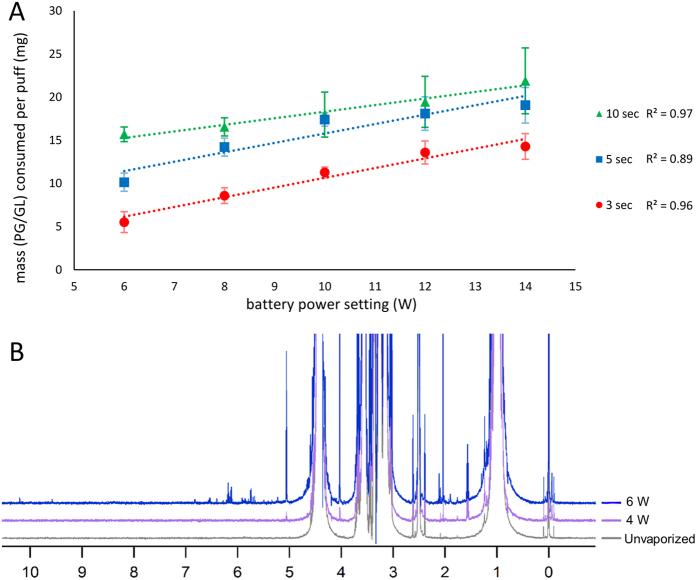
(**A**) PG and GLY consumption as a function of device power (W) as well as puff duration (sec), determined by mass. (**B**) ^1^H NMR spectra of unvaped control (bottom) PG/GLY solution along with aerosols derived from vaping PG/GLY at 4 W and 6 W showing product peaks increasing in number and intensity with increasing power.

**Figure 3:**
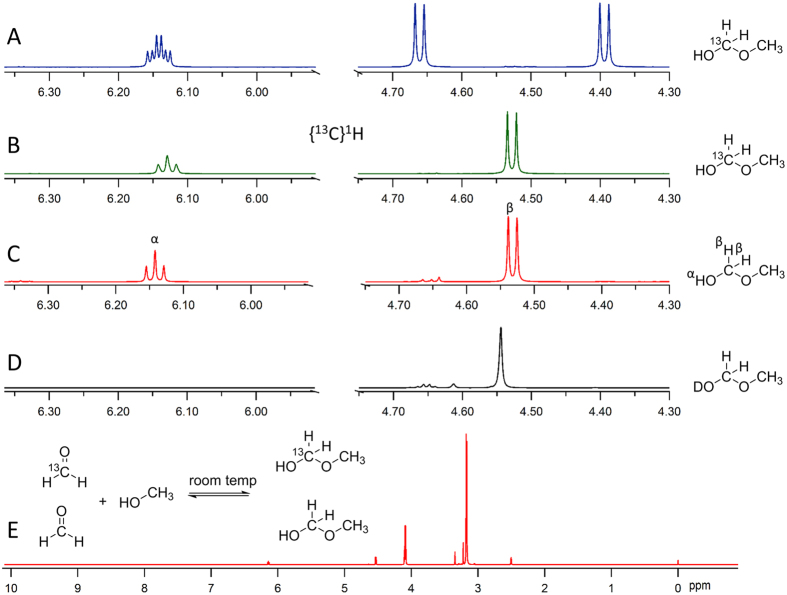
^13^C- and ^2^H-labeling study of model hemiformal production showing characteristic ^1^H NMR properties consistent with the previously reported NMR spectra of 1a and 1b^24^. (**A**) CH_3_OCH_2_OH exhibits analogous peak positions as well as the characteristic O-H proton splitting pattern corresponding to those for 1a and 1b. (**B**) The ^1^H-^13^C decoupled spectrum corresponds to that of the product formed by bubbling ^12^CH_2_O in CH_3_OH (spectrum **C**). (**D**) Upon addition of D_2_O the hydroxyl resonance at 6.14 ppm diminished and the methylene protons at 4.53 ppm collapse to a singlet. (**E**) The full ^1^H spectrum of CH_3_OCH_2_OH/CH_3_OH in DMSO-d_6_, for completeness.

**Figure 4:**
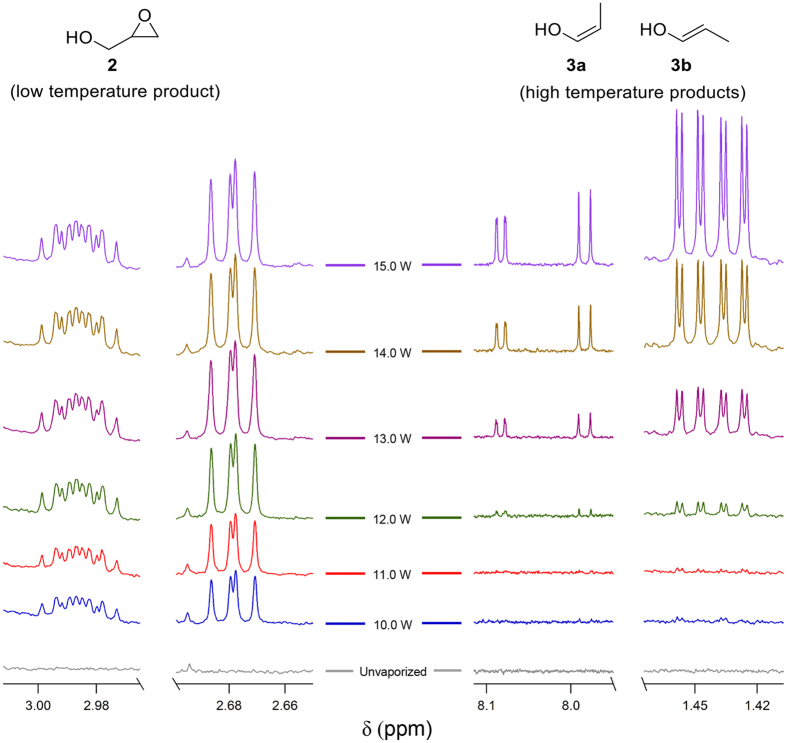
Wattage-dependent product formation. ^1^HNMR spectra were taken from a series of single puff samples of (PG/GL) collected in 1 W increments between 10W–15 W, showing the growing intensity of peaks associated with **2** (glycidol) and **3** (propanal enol isomers) as wattage is increased.

NMR spectra derived from aerosols produced at increasingly higher wattage settings revealed that, in addition to greater overall PG/GLY consumption and product formation at higher coil temperatures (Fig. 2), specific products arise at different settings. An expansion of the regions in the NMR containing the proton resonances of glycidol (**2)** as well as those of the *cis* and *trans* isomers of the propanal enols (**3**) is shown in Fig. 4. At 10 W, the peaks from **2** are present; however, the enol resonances are observable with adequate S/N only beginning at 12 W. This observation is in keeping with observations by Nef in 1904 when he described glycidol (**2)** as a “low temperature” product^23^. More recently, Laino and co-workers reported that the dehydration of glycerol to form **2** is the rate limiting step of a GLY degradation pathway^31^. Compound **2** has been shown to react with DNA^32^ It appears to be a relatively minor aerosol component based our studies to date (*vide infra*). Enols **3a** and **3b** have been previously found to persist for up to two weeks in dilute acetone at room temperature^33^. Their potential inhalation toxicology is not clear, though enol reactivity is well-known.

#### Aldehydes

**Figure 5:**
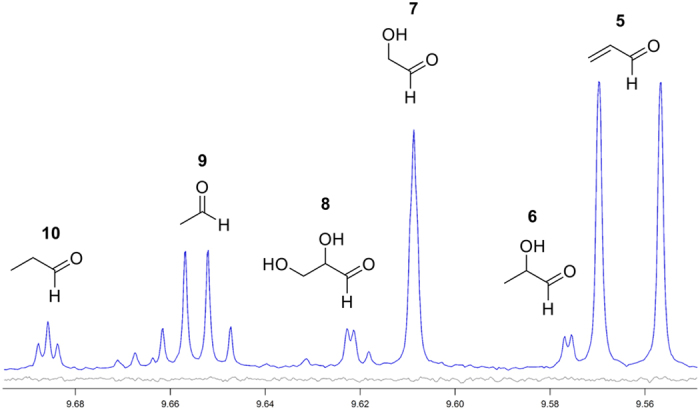
Expansion of the aldehyde region of a single puff-derived aerosol sample at 15 W. The relatively large peaks corresponding to acrolein (**5**) are noteworthy. Compound **6** = lactaldehyde; **7** = glycolaldehyde; **8** = glyceraldehyde; **9** = acetaldehyde; **10** = propanal.

An expansion of the aldehyde region of the ^1^H NMR spectrum of an aerosol generated from PG/GLY at 15 W, along with corresponding structure assignments, is shown in Fig. 5. Acrolein, compound **5,** has long been known as a decomposition product of GLY^20^. In fact, it is the target molecule of the most common qualitative chemical test for the detection of GLY^19^. Several routes have been proposed for its formation, including one from a recent study showing its formation via the GLY dehydration product **2**^31^. Acrolein is a well-known hazardous air pollutant^11^. Importantly, the doublet at 9.56 ppm corresponding to the aldehyde proton of **5** is highly prominent relative to those of the other products. This is significant because DNPH trapping cartridges used in prior chromatographic studies of e-cigarette aldehydes^13^ have been reported as unreliable for the determination of acrolein levels^34^, affording low recoveries. Prior reports of acrolein levels approaching those of other aldehydes have been characterized as having been attributed to overheating conditions^11^. Of the remaining aldehydes **6–10**, acetaldehyde (**9)** has garnered significant attention in e-cigarette aerosols because it is a possible human carcinogen (IARC Group 2B)^35^.

#### Reaction pathways

**Figure 6:**
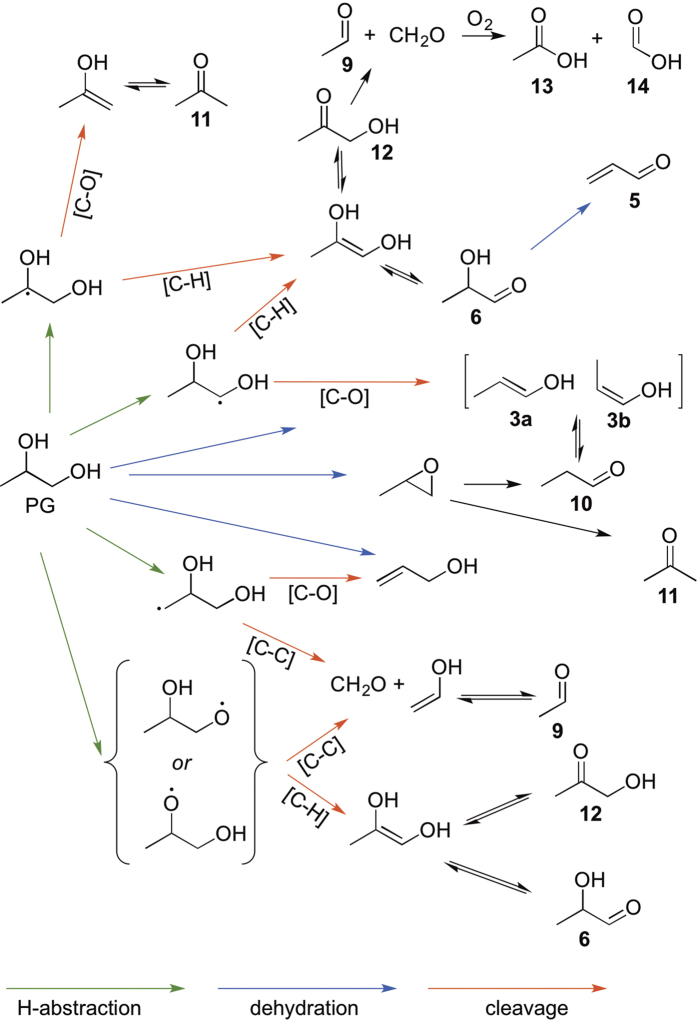
Aerobic Thermal Decomposition of Propylene Glycol.

**Figure 7:**
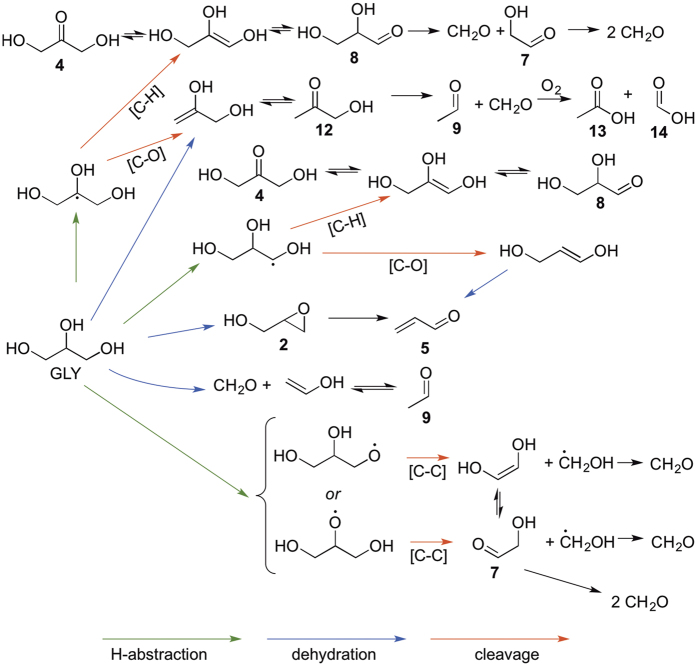
Aerobic Thermal Decomposition of Glycerol.

The reactions of PG and GLY under thermal conditions are predominantly dehydrations and oxidations (Figs 6 and 7). As mentioned previously, the conversion of PG to propanal (**10**) was reported in 1904 by Nef^23^. The oxidation of PG has also been previously shown to afford acetone (**11)**, acetaldehyde (**9)**, HCHO, acetol (**12)** and its tautomer lactaldehdye (**6)** along with dehydration product (**5)**^36^.

At the lowest device power setting, **12** was the major detectable aerosol product in the ^1^H NMR shown in Fig. 2. Compounds **12** and **6** can result from O-H proton abstraction via a higher temperature pathway owing to the relatively higher O-H bond dissociation energies. Cleavage of C–C bonds from the oxygen radicals would result in the formation of **13** and **14**, which can also form as oxidation or retro-aldol products of **12**. Compounds **13** and **14** were each observed herein at power settings between 10–15 W. A recent report on PG dehydration has shown that propylene oxide serves as an intermediate towards the formation of **10** and **11**, or can alternatively form allyl alcohol (**15**)^25^,^37^

GLY is oxidized to form dihydroxyacetone (**4**) and glyceraldehyde (**8)** by H atom abstraction, C–H bond cleavage and tautomerization. Compound **4** has been shown to possess genotoxic^38^ and mutagenic^39^ properties. Compound **12** is produced by the dehydration of GLY^40^,^41^. Compound **12** can further degrade to HCHO and acetaldehyde (**9)**^41^. Compound **8** can furnish **7** via a retro-aldol reaction. An ensuing retro-aldol affords HCHO, the abundance of which increases while that of **7** diminishes at high temperatures. Dehydration of GLY affords **2** and subsequently **5**^31^.

##### Physical factors modulating e-cigarette aerosol composition

As noted above, there is general agreement that increasing battery output, and thus the temperature of the heating coils, enhances the levels of PG/GLY degradation products in e-cigarettes. However, this does not clearly explain inter-laboratory differences in toxin levels. A recent report summarized HCHO (and other carbonyl) levels reported from five independent studies, each performed in 2014, and showed that the lowest HCHO range found was 3.2–3.9 ng/puff and the highest was 660–3400 ng/puff. The differences were attributed mainly to the different types of DNPH trapping cartridges used in each laboratory^11^. In addition to an inability to effectively recover specific compounds such as **5**, DNPH trapping columns were designed for gas-phase rather than aerosol compounds.

To begin to understand the unique differences that may arise between devices, such as a poor electrical connection or a manufacturing flaw, we investigated the vaping product yields of hydroxyacetone (**12**) and acetaldehyde (**9**) (Supporting Information) using three identical KangerTech^®^ Protank II clearomizers. Each clearomizer was equipped with a unique, identical 2.2 Ω coil and each fitted to one of three identical Innokin^®^ iTaste V4 batteries. Samples were run in triplicate. At power settings of 12 W and 15 W, one of the three devices afforded yields of both products that were several-fold higher compared to the other two devices. However, the mass of PG/GLY consumed from the reservoir of the outlier device during collection of the samples presented above was comparable to the other devices (Supporting Information).

**Figure 8:**
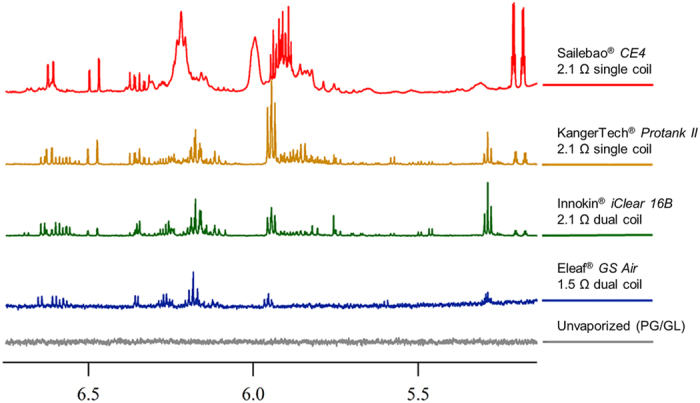
Comparison of (PG/GLY) decomposition at power settings of 15 W using clearomizers of differing configurations from various sources. An expanded region of the ^1^H NMR spectra of aerosolized samples of PG/GLY in addition to an unaerosolized sample (bottom). The red spectrum (top) shows aerosol products generated via an inexpensive Sailebao^®^ CE4 cartomizer and shows the highest abundance and diversity of degradation products. Now considered an outdated design, the CE4 clearomizers were purchased as a component of a “Starter Kit” in 2014, and are still widely available. Samples collected from more sophisticated devices typically produce spectra containing fewer peaks of lower intensity, as demonstrated by the spectra from samples vaped using the KangerTech^®^ Protank-II and the Innokin^®^ iClear 16B, plotted in orange and green, respectively. Expensive, more current devices such as the Eleaf^®^ GS Air produce aerosolized samples showing relatively diminished product peak intensities as well as fewer degradation product peaks. While the extent of (PG/GLY) degradation varies between models, it is not unique to any one design.

One issue that will affect the degree of product degradation is the efficiency of latent heat transfer from the heating coils to the e-liquid. The bottom coil configuration of the clearomizer used herein is more efficient than the typically shorter top load coils of relatively older clearomizers and cartomizers. In addition, multiple coils should be the most efficient at dispersing heat over the entire solvent volume, and would be expected to afford the least PG/GLY degradation. Figure 8 shows a representative comparison of expansions of the NMR spectra of aerosols produced by each of four clearomizers. All aerosols contained **1a** and **1b** at 6.2 ppm; however, in the case of the CE4 clearomizer (top spectrum) peak broadening was significant due to enhanced exchange due to the high concentration. It is apparent by visual inspection, as expected, that the top coil clearomizer afforded the most degradation products, whereas one of the dual coil clearomizers afforded the least. The issue of heat transfer is thus not only significant in designing safer devices, but will contribute to inter-laboratory variability in reported e-cigarette product levels. For instance, one would expect that differences in coil gauges, the number of turns and morphological defects would be among factors influencing heat transfer and product formation.

**Figure 9:**
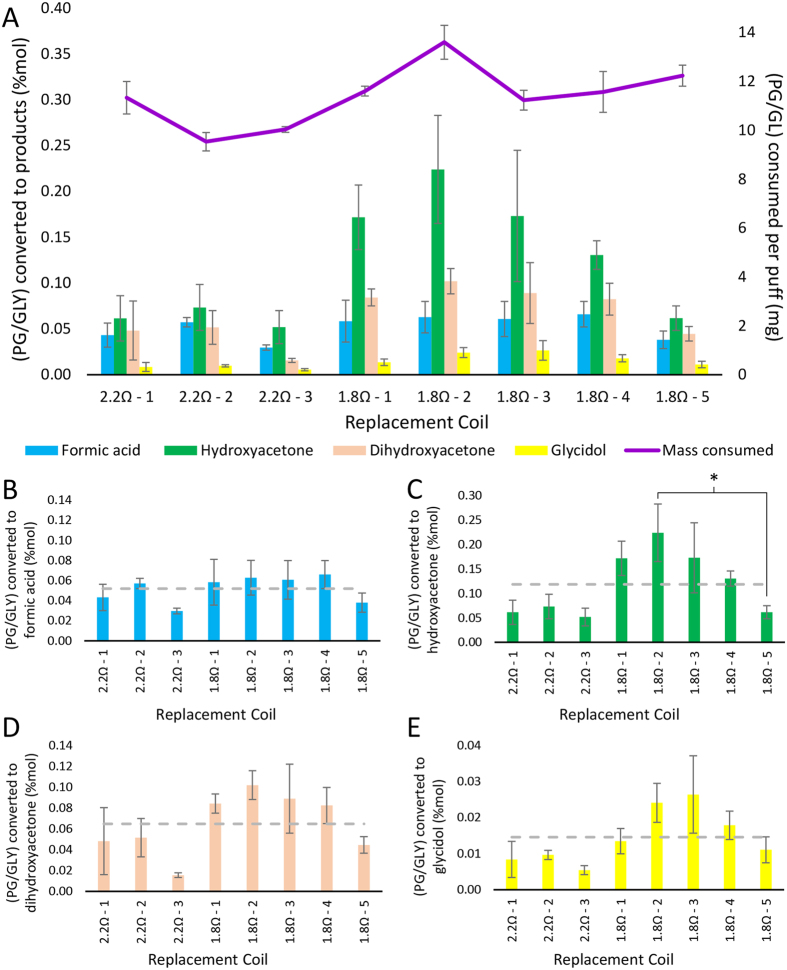
Individual heating elements of the same design and manufacture, identical in appearance and packaged together as replacement units, can demonstrate wide variation in the abundance and profile of decomposition products observed in samples collected under controlled conditions. Depicted above are results from aerosolized samples of PG/GLY collected under fixed conditions from the KangerTech^®^ Protank-II clearomizer, varying only the replaceable single-coil heating element. Three single-puff samples were collected at a modest power setting of 10 W from eight different replacement coils, three of which were labeled 2.2 Ω resistance by the manufacturer, while the other five were labeled 1.8 Ω resistance. (**A**) The intensity of NMR signals from several degradation products were compared by relative integration to the intensity of un-degraded PG/GLY peaks; the relative intensity of the thermal degradation products formic acid (Plot **B**, compound **14**), hydroxyacetone (Plot **C**, compound **12**), dihydroxyacetone (Plot **D**, compound **4**), and glycidol (Plot **E**, compound **2**) are plotted as percentages of the intensity of residual (PG/GLY). The average value among the eight replacement coils is plotted as a grey dashed line in plots **B–E**. All error bars denote a 90% confidence interval (n = 3). The asterisk (*) in plot **C** denotes p < 0.05 as determined by a two-variable, unpaired t-test.

To investigate the role of inter-coil differences in product formation, we examined eight replacement coils for a clearomizer. Three or more coils were tested at each of two resistances for their effect on PG/GLY product levels, using the same battery. However, there was some statistically significant variability in the product levels observed from different coils with the same resistance values (Fig. 9). Consistent trends were also observed, such as glycidol (**2**) formed at the lowest levels as compared to the other three products shown. Hydroxyacetone (acetol, **12**) was the major product using the lower resistance coils. Dihydroxyacetone (**4**) was observed as a relatively abundant product formed via the majority of the eight coils. Overall, the fact that there are statistical differences (t_critical_ = 4.303, DF = 2, p < 0.05) in product levels using the same device and power levels while varying only coils of the same model and resistance levels, exacerbates the challenges inherent in controlling device and inter-laboratory consistency.

##### Chemical factors modulating e-cigarette aerosol composition

Many of the reaction sequences shown in Figs 6 and 7 have rate limiting steps with activation energies well over 50 kcal/mol determined under pyrolysis conditions. For example, Laino *et al*. reported that heating PG to 537 °C for 30 s without O_2_ led to a nearly 99.9% recovery of unreacted compound, and that GLY had very similar thermal stability^37^. However, since e-cigarettes are used under aerobic conditions, the presence of O_2_ will play a significant role in promoting oxidation.

Although the chemistry of PG and GLY has been studied for centuries, there is still a relative lack of understanding of their reactions under aerobic conditions, as recently noted by Diaz^36^ and Hemings *et al*.^42^ The evidence reported to date, however, clearly shows that O_2_ initiates the thermal degradation of PG and GLY at significantly lower temperatures as compared to anaerobic (pyrolysis) conditions. Diaz *et al*. demonstrated that O_2_-promoted hydrogen abstraction from PG (Fig. 6) to form products derived from carbon-centered radicals at temperatures as low as 127 °C over the course of 6–14 seconds in the presence of O_2_ 36 No reaction was observed under the same conditions under an inert atmosphere.

Based on the available evidence, GLY is relatively more stable to oxidation as compared to PG, with polymerization and decomposition reportedly initiating at 200 °C^43^. At temperatures close to the boiling point of GLY (290 °C), Sabatier and Gaudin produced glyceraldehyde **(8)** as a main intermediate in the formation of CO_2_ and ethanol, **9** and **10**^44^. Stein and co-workers reported no evidence of GLY degradation at temperatures of up to 200 °C in the presence of O_2_, but observed discoloration after heating samples to 250 °C^45^. They ascribed H-atom abstraction as the initial free radical reaction (Fig. 7), analogous to that observed for PG by Diaz^36^, as leading to the production of acrolein, acetaldehyde, and formaldehyde.

In order to confirm that O_2_ promotes product formation, we compared product yields obtained under ambient vs. reduced-O_2_ conditions. An obvious decrease in the intensity of many ^1^H NMR peaks – corresponding to decomposition products was observed when samples of aerosolized PG/GLY were collected in a sealed glove-bag that had been flushed with N_2_ (Supporting Information). Interestingly, specific product yields associated with decomposition products in the anaerobic spectra, including glycolaldehyde (**7**) and hydroxyacetone (**12**) were relatively less influenced by the absence of oxygen as compared to the formation of the other products, and were the most abundant decomposition products in the reduced-O_2_ derived samples (Supporting Information).

In addition to oxidation, the other main thermal reaction of PG and GLY is dehydration. It is well-known that dehydration reactions are catalyzed by acid. In 1985 Rossiter described the degradation of aqueous glycol solutions, including PG, in the presence and absence of air and metals, noting that the acidic products that form as a result of thermal breakdown decrease the solution pH, catalyzing further degradation^46^. More recently, Nimlos and co-workers showed that activation energies for the dehydration of neutral GLY ranged from 65.2–79.5 kcal/mol, but were lowered to 20–25 kcal/mol when GLY was protonated^47^. In addition to protonation, metal catalysis is well-known to promote PG and GLY reactivity. Laino has also modeled the interactions of PG and GLY with various metal surfaces which would have relevance to e-cigarettes due, for instance, not only to interaction with device components but also with metals and metal nanoparticles found in e-cigarette aerosols^48^.

Although acidic products have been observed in e-cigarette aerosols (Fig. 2b; the peak downfield of 10 ppm, for example), their contribution to the overall pH will not generally be as significant as relatively more abundant acidic additives, such as certain flavorants. The study of the effects of acidic flavorants on product profiles during vaping is currently under investigation in our laboratories.

Limitations of this study include the relative lack of sensitivity of NMR as compared, for instance, to mass spectrometry. Moreover, the NMR spectra of e-cigarette aerosols have overlapping peaks, hindering complete product profiling. In addition, studies were performed on single puff samples, which depresses the average temperature of the heating coils as compared to multiple puff real-world usage, thereby likely underestimating realistic aerosol product levels. However, the primary goals of this investigation were (i) analytical target discovery, namely identifying aerosol components that were overlooked or under-investigated to date in the e-cigarette field, as well as (ii) clarifying the reasons for some of the discrepancies in the results reported by various labs, and (iii) an extensive description of the chemical pathways of PG and GLY degradation in the context of e-cigarette usage.

## Conclusion

In this investigation we used NMR for e-cigarette aerosol product identification with no aerosol sample processing, apart from dilution in DMSO-d_6_. As had been proposed, a main finding was that the e-cigarette solvents PG and GLY afford products that are fully consistent with prior studies of their pyrolysis and combustion. In addition, the results herein suggest NMR as a viable alternative to DNPH trapping cartridges for monitoring challenging, reactive toxins such as acrolein. Finally, (i) the sensitivity of PG and GLY to thermal oxidation, (ii) the catalysis of their dehydration reactions by acids and/or metals, and (iii) the variability in the heat transfer efficiencies of individual clearomizers and heating coils should be taken into account when considering strategies to minimize toxin production and inter-laboratory inconsistencies in evaluating these devices.

## Additional Information

**How to cite this article**: Jensen, R. P. *et al*. Solvent Chemistry in the Electronic Cigarette Reaction Vessel. *Sci. Rep.* **7**, 42549; doi: 10.1038/srep42549 (2017).

**Publisher's note:** Springer Nature remains neutral with regard to jurisdictional claims in published maps and institutional affiliations.

## Supplementary Material

Supplementary Information
