# Supplementary material for: Solvent Chemistry in the Electronic Cigarette Reaction Vessel
Source: Sci Rep. 2017 Feb 14;7:42549. doi: 10.1038/srep42549 (PMC5307352; doi:10.1038/srep42549)
Supplement: Supplementary Information [file srep42549-s1.pdf]

Solvent Chemistry in the Electronic Cigarette Reaction Vessel

R. Paul Jensen, Robert M. Strongin and David H. Peyton*

Department of Chemistry, Portland State University, Portland, Oregon 97207

Supporting Information

Materials and Methods S2
Quantitative Sample Collection S2
Quantification of Hydroxyacetone S3
Quantification of Acetaldehyde S10
Identification and Confirmation of Decomposition Products S10-S25
The Effect of Oxygen on Solvent Decomposition S27-S29

Table S1. Conditions and Consumption of Quantitative Samples S3
Table S2. Quantification of Hydroxyacetone S4
Table S3. Quantification of Acetaldehyde S6
Table S4. Conditions and Consumption of Aerobic/Anaerobic Samples S26

Figure S1. Increased Consumption of (PG/GL) with Increased Operating Power and Puff Duration S3
Figure S2. Hydroxyacetone Values Averaged from Three Devices S5
Figure S3. Hydroxyacetone Values From Each Device, Separated by Puff Duration S6
Figure S4. Acetaldehyde Values Averaged from Three Devices S8
Figure S5. Acetaldehyde Values From Each Device, Separated by Puff Duration S9
Figure S6. Confirmation of Decomposition Product Identity by Minute Addition S11
Figure S7. ^{1}H NMR Assignment of Hydroxyacetone and Confirmation in Vaporized PG/GL S12
Figure S8. ^{1}H NMR Assignment of Acrolein and Confirmation in Vaporized PG/GL S13
Figure S9. ^{1}H NMR Assignment of Glycidol and Confirmation in Vaporized PG/GL S14
Figure S10. ^{1}H NMR Assignment of Acetaldehyde and Confirmation in Vaporized PG/GL S15
Figure S11. ^{1}H NMR Assignment of Dihydroxyacetone and Confirmation in Vaporized PG/GL S16
Figure S12. ^{1}H NMR Assignment of Glyceraldehyde and Confirmation in Vaporized PG/GL S17
Figure S13. ^{1}H NMR Assignment of Glycolaldehyde and Confirmation in Vaporized PG/GL S18
Figure S14. ^{1}H NMR Assignment of Allyl Alcohol and Confirmation in Vaporized PG/GL S19
Figure S15. ^{1}H NMR Assignment of Acetic Acid and Confirmation in Vaporized PG/GL S20
Figure S16. ^{1}H NMR Assignment of Formic Acid and Confirmation in Vaporized PG/GL S21
Figure S17. ^{1}H NMR Assignment of Propanal and Confirmation in Vaporized PG/GL S22
Figure S18. Tautomerization of *E,Z*-prop-1-ene-1-ol to Propanal in Vaporized PG/GL S23
Figure S19. ^{1}H NMR Assignment of Acetone and Confirmation in Vaporized PG/GL S24
Figure S20. ^{1}H NMR Assignment of Lactaldehyde and Confirmation in Vaporized PG/GL S26
Figure S21. ^{1}H NMR Assignments of various FRA hemiacetals of formaldehyde with PG and GLY (GL) in vaporized PG/GL S27
Figure S22. PG/GL Vaporized Under Anaerobic and Aerobic Conditions S28
Figure S22. Quantification of Hydroxyacetone and Glycolaldehyde in Anaerobic and Aerobic Samples S28
Figure S23. Averaged Values of Hydroxyacetone and Glycolaldehyde in Anaerobic and Aerobic Samples S29

EXPERIMENTAL SECTION

Materials and Methods

All solvents and reagents were purchased from Sigma-Aldrich and used as received. NMR spectra were collected using Bruker Avance III 400 or 600 MHz NMR spectrometers. The mixture of (PG/GL) used in this study was made by adding 18.4180 g of glycerol and 15.2180 g of propylene glycol (both USP grade, Sigma-Aldrich) together into a 100 mL bottle on an analytical balance, then capping and shaking the mixture for an extended period. The mixture is analyzed by ^{1}H NMR spectroscopy and confirmed as 1:1 molar ratio by relative integration. This (PG/GL) mixture may be added to the e-liquid reservoir of a clearomizer using a Pasteur pipet.

Puffs of vaporized (PG/GL) were drawn through 1-inch-long, 18 gauge disposable needles into 1500 μL glass vials containing 600 μL of solvent for NMR analysis. These septa-capped vials were weighed both before and after collection of each sample to determine the mass of the sample collected. Collected samples were vortexed and transferred to a 5 mm NMR tube for analysis. Quantitative ^{1}H NMR experiments were conducted in DMSO-D_6 (99.9%, Cambridge Isotope) containing 0.5 % v/v TMS as a chemical shift reference. For quantification by relative integration, a 1.0 mM solution of an internal standard (1,2,4,5-tetrachloro-3-nitrobenzene, M.W. = 260.89 g/mol) was made by 1:10 dilution from a 10.0 mM stock solution made by dissolving 0.1304 grams of the solid in a 50 mL volumetric flask. Samples were analyzed by ^{1}H NMR spectroscopy at 599.80 MHz using 30-degree flip angles and long inter-scan delays to ensure full relaxation. Relative integration against the internal standard was used to establish a concentration of decomposition products, which was used to determine the mass of each product within the 600 μL sample. The calculated mass was then taken as a ratio of the total mass of vaporized (PG/GL) collected in the sample to determine the mass percent (%w/w) of the product in the sample.

Quantitative Sample Collection. The samples collected and analyzed in this experiment are outlined in Table S1. Each sample consisted of three 50 mL puffs of (PG/GL) vaporized in ascending order at 6, 8, 10, 12, and 14 Watts over the duration of 3, 5, and 10 second puffs for a total of 15 samples composed of 45 puffs. The clearomizer was removed from the e-cigarette and weighed on an analytical balance before and after each of the three puffs taken per sample in order to determine the mass of (PG/GL) consumed from the reservoir during each puff. In this experiment, three identical KangerTech® *Protank II* clearomizers were used to collect triplicate sets of samples. Each clearomizer was equipped with a unique, identical 2.2 Ω coil and each fitted to one of three identical Innokin® *iTaste V4* batteries. These triplicate sets of samples taken from identical devices are intended to account for the unique differences that may arise between devices, such as a poor electrical connection or a manufacturing flaw. Quantitative values determined by analysis of samples collected from these devices (designated *A*, *B*, and *C*) are presented below, first averaged together, then disjointed to show the variation between devices. The masses of (PG/GL) consumed per puff shown in Table S1 are the average +/- standard deviation of nine identical puffs (three each taken from three devices). The consumption data from Table S1 is plotted in Figure S1, representing 45 samples composed of 135 puffs, taken from three different devices.

Table S1 – Conditions and Consumption of Quantitative Samples.

Collection Order (Sample #)	E-cigarette Power (Watts)	Duration of 50 mL puff (seconds)	Average (n=9) Mass of (PG/GL) consumed from reservoir per puff (grams)	Error (S.D. n=9)
**1**	6	3	5.5	1.2
**2**	6	5	10.1	1.0
**3**	6	10	15.7	0.8
**4**	8	3	8.6	0.9
**5**	8	5	14.2	1.0
**6**	8	10	16.6	1.0
**7**	10	3	11.3	0.6
**8**	10	5	17.4	0.8
**9**	10	10	18.0	2.6
**10**	12	3	13.6	1.3
**11**	12	5	18.1	2.0
**12**	12	10	19.5	3.0
**13**	14	3	14.3	1.5
**14**	14	5	19.1	2.1
**15**	14	10	21.9	3.8

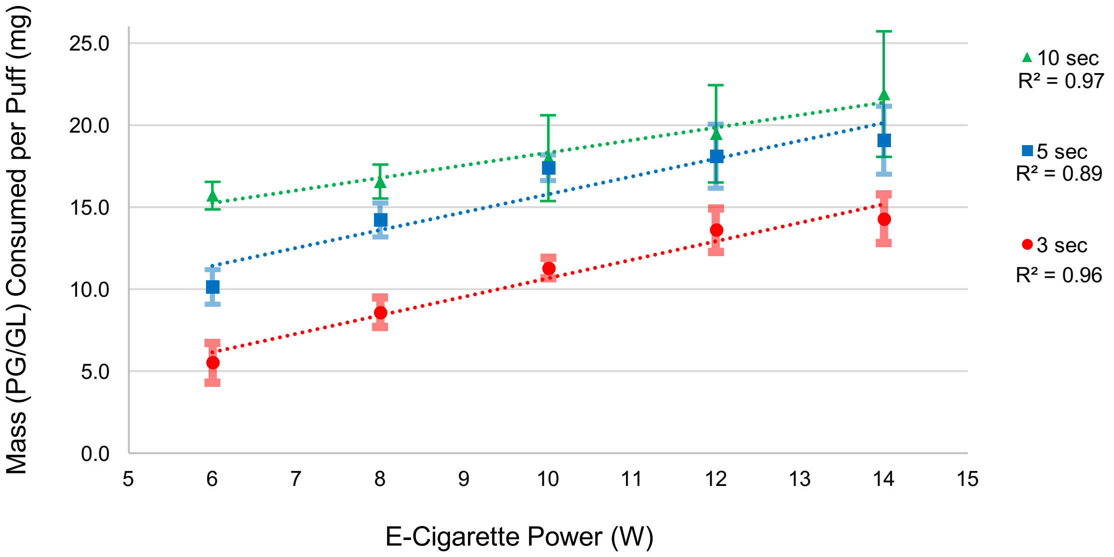

Figure S1 *– Increased Consumption of (PG/GL) with Increased Operating Power and Puff Duration.*

Figure S1 shows a general increase in the mass of (PG/GL) consumed from the reservoir with increases in both operating power and puff duration – variables that likely correlate with operating temperature. A similar relationship was hypothesized to exist between increased operating temperature and the extent of sample degradation.

Quantification of Hydroxyacetone

Table S2 shows the concentrations of hydroxyacetone in collected samples as measured by relative integration against an internal standard, along with calculated values of the mass percent of hydroxyacetone in the sample +/- the standard error in the measurement – these values are plotted in Figure S2. The error terms are large relative to the calculated sample percentage, reflecting a high degree of variability between devices used in this experiment – this can be observed in Figure S3, which presents the calculated %w/w of hydroxyacetone in the samples generated by each of the three identical devices (A, B, and C) used in this experiment.

Table S2 – Quantification of Hydroxyacetone.

	Power (W)	Average [hydroxyacetone] (mM)	Average % hydroxyacetone (w/w)	error
3 sec	6	0.020	0.031	0.015
	8	0.090	0.080	0.062
	10	0.393	0.221	0.124
	12	0.920	0.634	0.404
	14	1.160	0.643	0.427
5 sec	Power (W)	Average [hydroxyacetone] (mM)	Average % hydroxyacetone (w/w)	error
	6	0.090	0.036	0.022
	8	0.383	0.157	0.112
	10	0.833	0.403	0.260
	12	1.117	0.578	0.413
	14	1.397	1.012	0.695
10 sec	Power (W)	Average [hydroxyacetone] (mM)	Average % hydroxyacetone (w/w)	error
	6	0.450	0.133	0.098
	8	1.020	0.331	0.184
	10	1.297	1.196	0.742
	12	1.777	0.923	0.587
	14	1.710	1.452	1.026

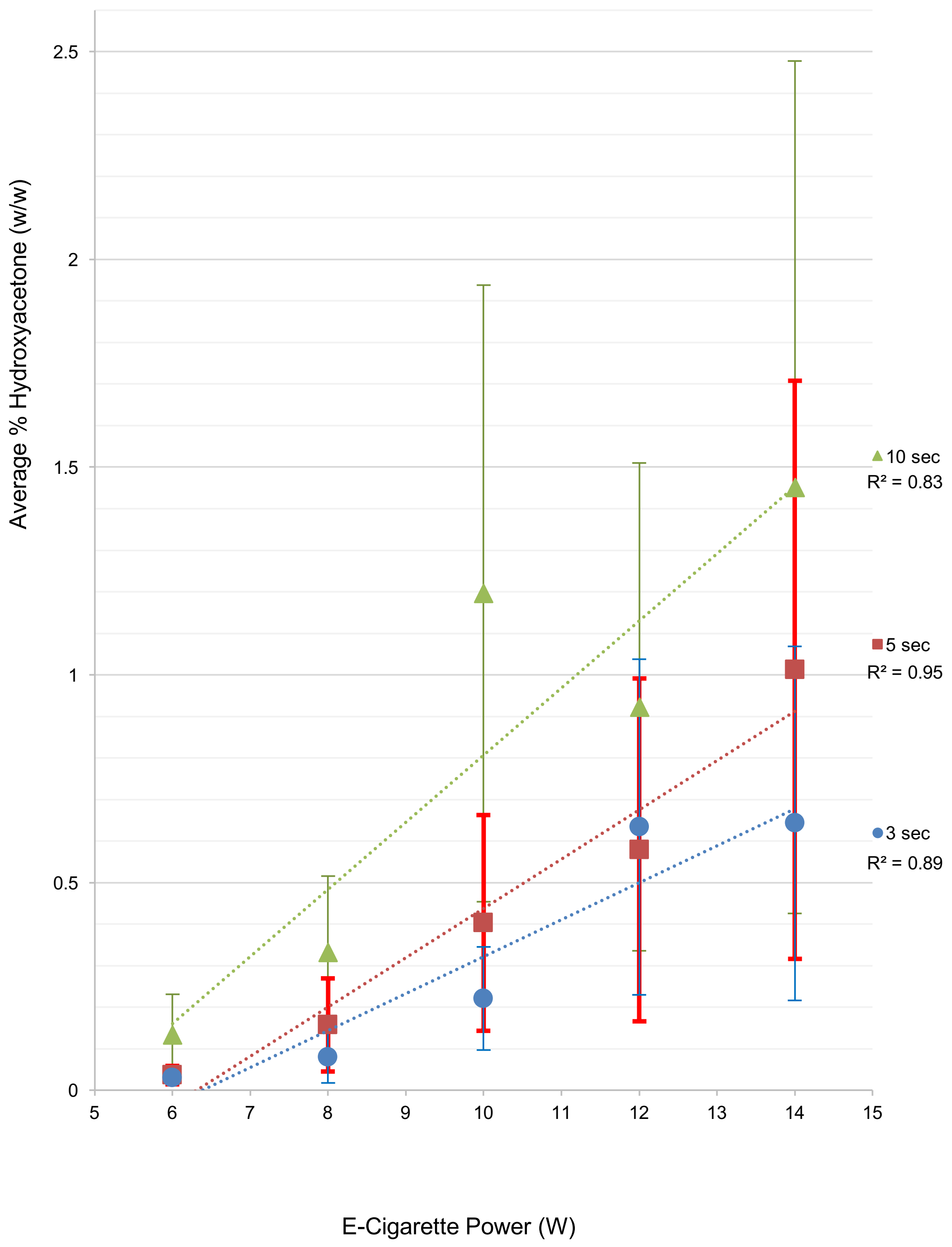

Figure S2 –Hydroxyacetone Values Averaged from Three Devices.

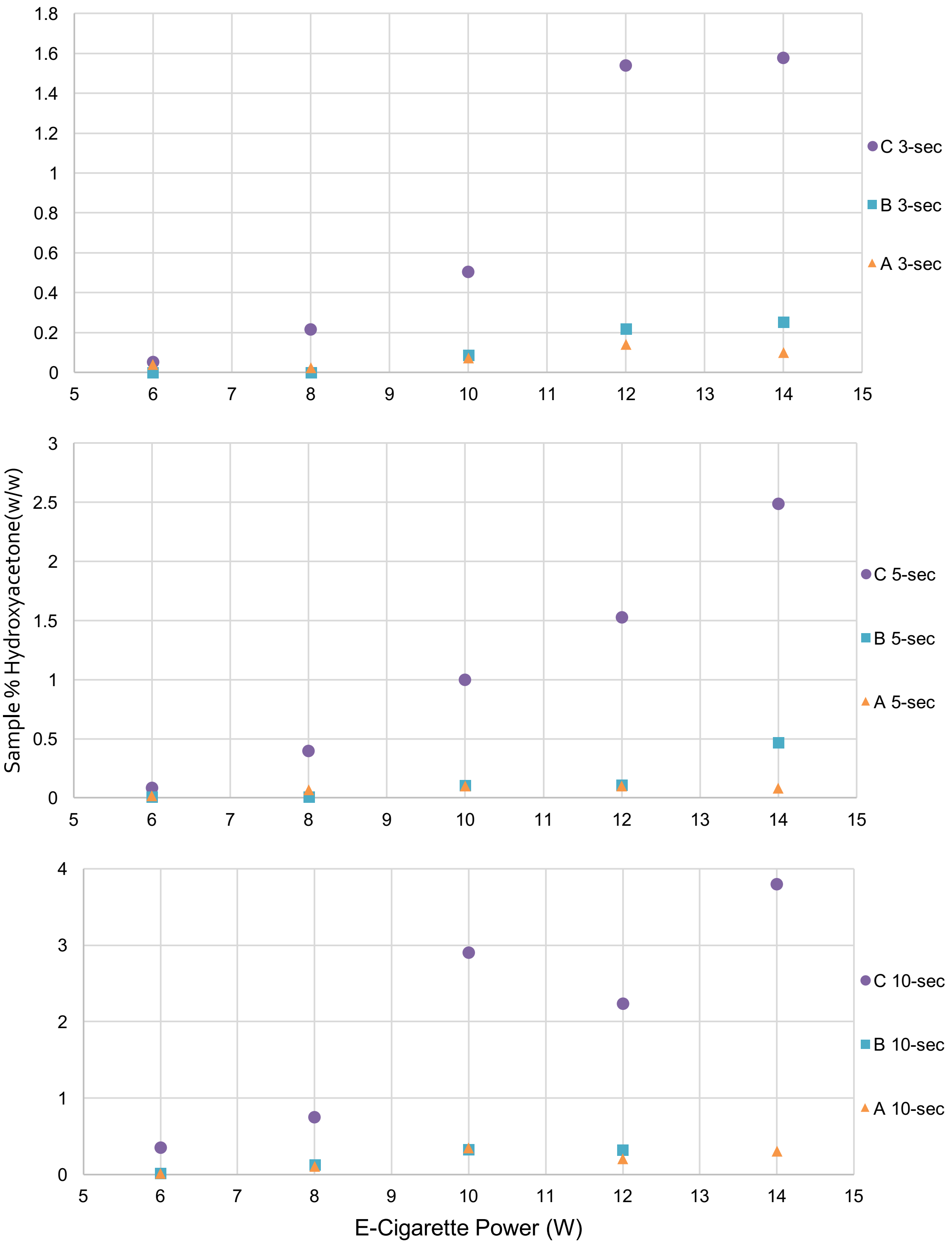

Figure S3 –Hydroxyacetone Values From Each Device, Separated by Puff Duration.

Figure S3 shows that in each of the three conditions of puff duration (3, 5, and 10 seconds), the samples vaporized using e-cigarette device *C* contain significantly more hydroxyacetone than the samples collected from the other two devices, especially at high power levels. The extremely high levels of decomposition observed in samples vaporized using device *C* is the source of the large error terms observed in Table S2 and Figure S2. The mass of (PG/GL) consumed from the reservoir of device *C* during collection of the samples presented above is unremarkable in comparison with the other devices,

indicating that there has been no failure to keep the wick and coil wetted with liquid during sample collection.

Table S3 – Quantification of Acetaldehyde.

	Power (W)	Average [acetaldehyde] (mM)	Average % acetaldehyde (w/w)	error
3 sec	6	0.003	0.003	0.002
	8	0.013	0.007	0.006
	10	0.130	0.042	0.032
	12	1.037	0.427	0.345
	14	1.620	0.557	0.443
	Power (W)	Average [acetaldehyde] (mM)	Average % acetaldehyde (w/w)	error
5 sec	6	0.023	0.005	0.004
	8	0.397	0.097	0.072
	10	1.287	0.376	0.278
	12	2.087	0.680	0.548
	14	2.323	1.124	0.868
	Power (W)	Average [acetaldehyde] (mM)	Average % acetaldehyde (w/w)	error
10 sec	6	0.130	0.022	0.018
	8	0.510	0.098	0.053
	10	1.477	0.730	0.406
	12	4.163	1.671	1.351
	14	3.117	2.042	1.665

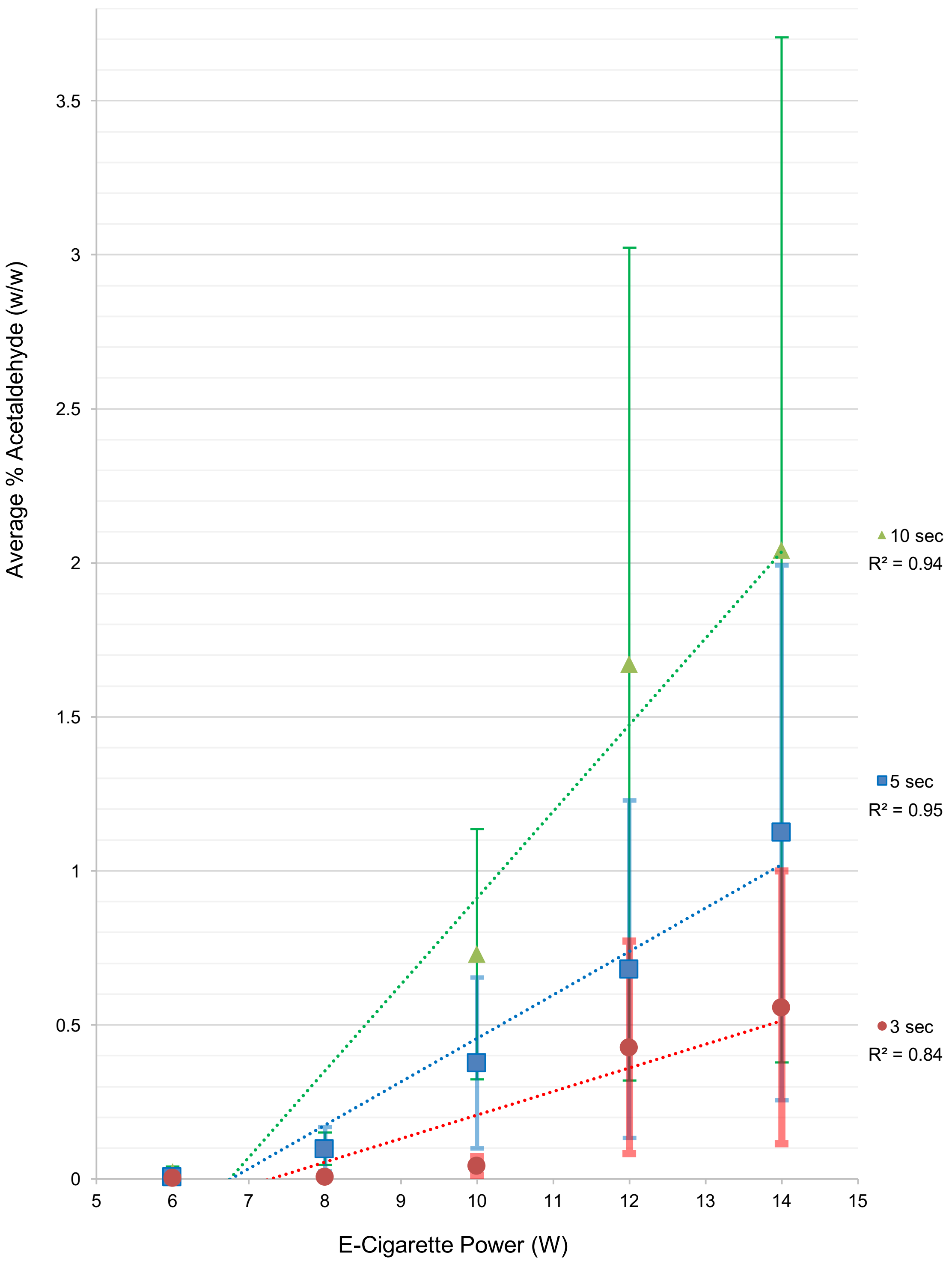

Figure S4 –Acetaldehyde Values Averaged from Three Devices.

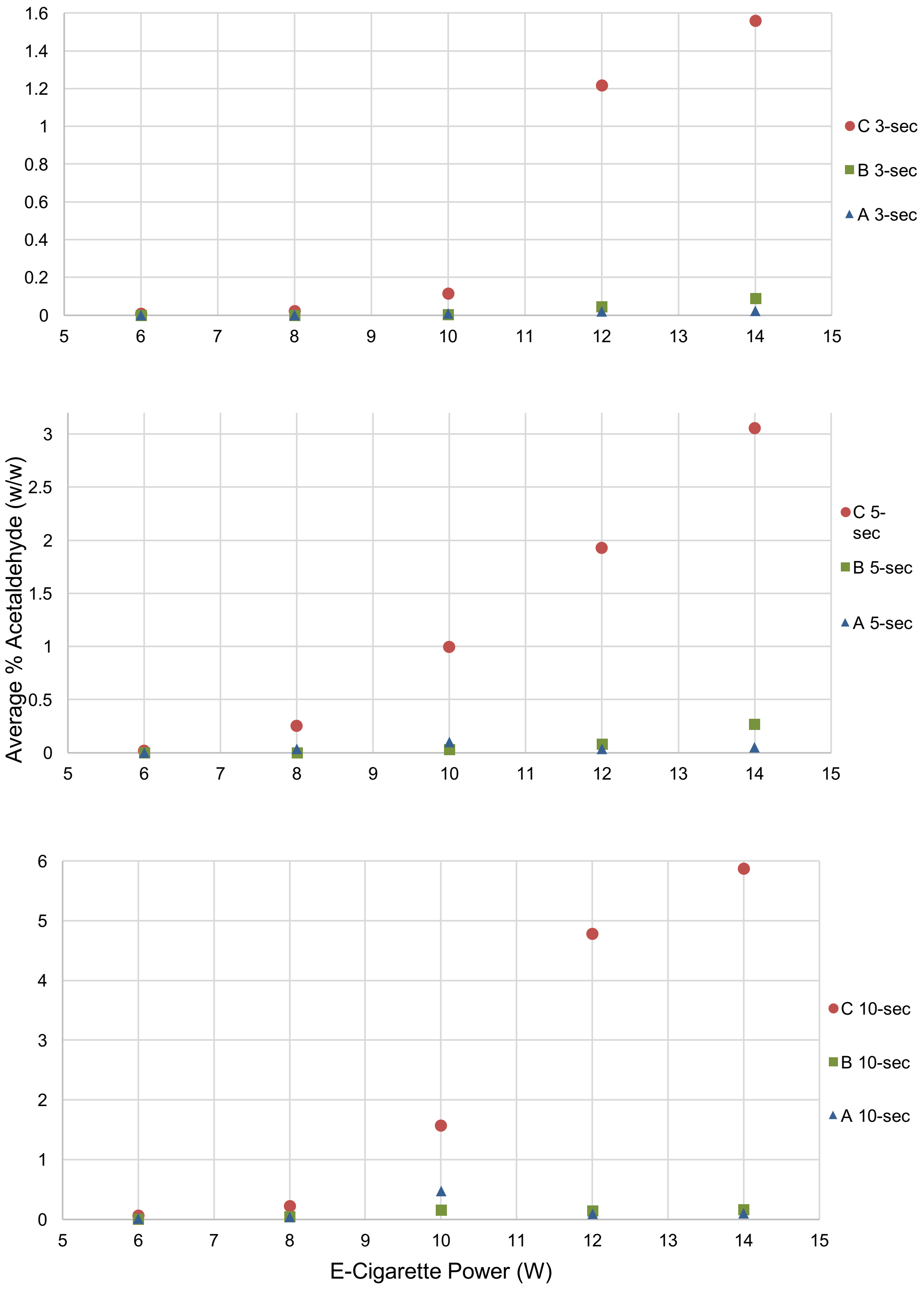

Figure S5 –Acetaldehyde Values From Each Device, Separated by Puff Duration.

Quantification of Acetaldehyde
Table S3 shows the concentrations of acetaldehyde in collected samples as measured by relative integration against an internal standard, along with calculated values of the mass percent of acetaldehyde in the sample +/- the standard error in the measurement, which are plotted in Figure S4. The error terms are again large relative to the calculated sample percentage, reflecting a high degree of variability between devices used in this experiment – this can be observed in Figure S5, which presents the calculated % acetaldehyde generated by each of the three devices (*A*, *B*, and *C*) used in this experiment.

As was the case with hydroxyacetone, Figure S5 shows that in each of the three conditions of puff duration (3, 5, and 10 seconds), the samples vaporized using e-cigarette device *C* contain significantly more acetaldehyde than the samples collected from the other two devices. The extremely high levels of decomposition observed in samples vaporized using device *C* is the source of the large error terms observed in Table S3 and Figure S4.

Identification and Confirmation of Decomposition Products
Vaporized samples of (PG/GL) were collected directly into aliquots of DMSO-D_6 contained within pre-weighed glass vials capped with PTFE septa; during quantitative experiments, the solvent was carefully prepared with an internal standard (1,2,4,5-tetrachloro-3-nitrobenzene at 1.0 mM) for quantification by relative ^{1}H integration. After collection, the vials were weighed to determine the mass of samples collected, and the solutions were transferred to 5 mm glass NMR tubes for analysis. At high magnetic field-strengths, one-dimensional ^{1}H analysis is straightforward with as little as one single 50 mL puff of (PG/GL) consisting of 5-10 mg, although many scans may be required to collect a spectrum with an acceptable signal-to-noise ratio. Samples consisting of three to ten 50 mL puffs of (PG/GL) significantly cut down on acquisition time and allow for much easier one-dimensional ^{13}C, COSY, TOCSY, NOESY, ^{1}H-^{13}C HSQC and HMBC experiments. Collecting too many puffs of vaporized (PG/GL) may result in overly-concentrated samples and diminished quality of observed ^{1}H signals; an abundance of glycerol makes samples viscous and increases the rate of exchange, diminishing peaks and collapsing the observed splitting of exchangeable protons such as hydroxyls. When studying glycerol by ^{1}H NMR spectroscopy, increased signal intensity is bought at the expense of fine detail.

After a compound has been speculatively identified by chemical shift, peak shape, coupling constants, and relative integration, the identity of the product is confirmed by adding a very small portion of the pure compound obtained from a commercial source to the sample and observing a small increase in the intensity of the peaks under investigation. If sufficiently little of the pure compound is added, the results of such an experiment can provide confirmation of identity; if too much is added, the addition of large resonances to the spectrum will overwhelm the peaks under investigation and no confirmation can be made.

The data generated by this *minute addition* experiment is demonstrated in Figure S6, showing a successful confirmation of acetone in a sample of vaporized (PG/GL). In green, plotted across the top, is the ^{1}H NMR spectrum of pure acetone diluted in DMSO-D_6. In grey, plotted across the bottom, is the ^{1}H NMR spectrum of unvaporized (PG/GL), which contains no peaks in the acetyl region. In blue is plotted the ^{1}H NMR spectrum of a sample of vaporized (PG/GL) containing new peaks in the acetyl region corresponding to decomposition products. When a very small amount of acetone is added to the sample of vaporized (PG/GL), one of the peaks in the acetyl region grows, while the others remain at the same intensity, as shown in the spectrum plotted in the red dotted line. Figures such as these are presented here for a number of decomposition products.

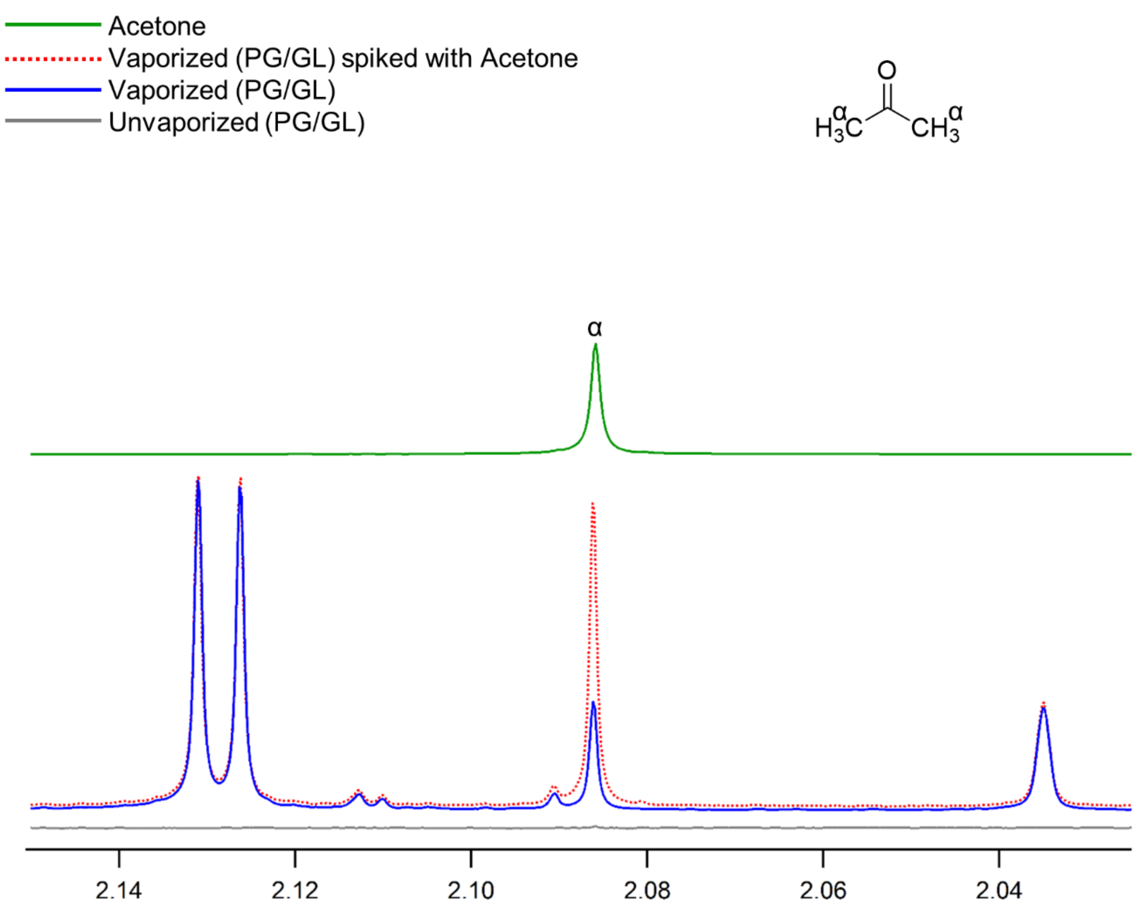

Figure S6 – Confirmation of Decomposition Product Identity by Minute Addition.

Hydroxyacetone

Hydroxyacetone is often the most populous decomposition product present in samples of vaporized (PG/GL), being a primary dehydration product of glycerol *and* a primary oxidation product of propylene glycol. When samples are vaporized at low temperatures by using low power settings and short puff durations, this compound is sometimes the only degradation product detected. This is typified in Figure 2 in the text, which shows a ^{1}H NMR spectrum of (PG/GL) vaporized at 4 Watts containing little evidence of decomposition products save a few small peaks corresponding to hydroxyacetone. As the power level and/or puff duration used to collect vaporized (PG/GL) samples increases, hydroxyacetone continues to increase in intensity; only rarely is it outnumbered by any other single decomposition product.

The compound has a relatively simple structure to solve and confirm using ^{1}H and ^{13}C NMR data. Having a ketone (and thus no protons) at the β-carbon, the resonance signal of the adjacent acetyl group (s, 2.04 ppm) remains a Lorentzian singlet located near the signals of other acetyl compounds such as acetone and acetic acid. The two equivalent methylene protons (d, 4.02 ppm) are split into a doublet by the adjacent hydroxyl proton (t, 5.06 ppm), which is likewise split into a triplet by the methylene protons. The chemical shift of the methylene doublet falls between the large resonances of the hydroxyl and alkyl protons of un-degraded (PG/GL) and is un-occluded in samples of vaporized (PG/GL) when observed at high frequency. The hydroxyl proton is labile to hydrogen-deuterium exchange experiments, disappearing from the spectrum while the methylene doublet collapses into a singlet. The chemical shift of the hydroxyl proton is very susceptible to changes in temperature or concentration. The acetyl resonance, being un-split and composed of three equivalent protons, is much taller than the other signals. The assigned ^{1}H NMR spectrum of hydroxyacetone is shown in Figure S7, along with confirmation of the compound in vaporized (PG/GL) by minute addition.

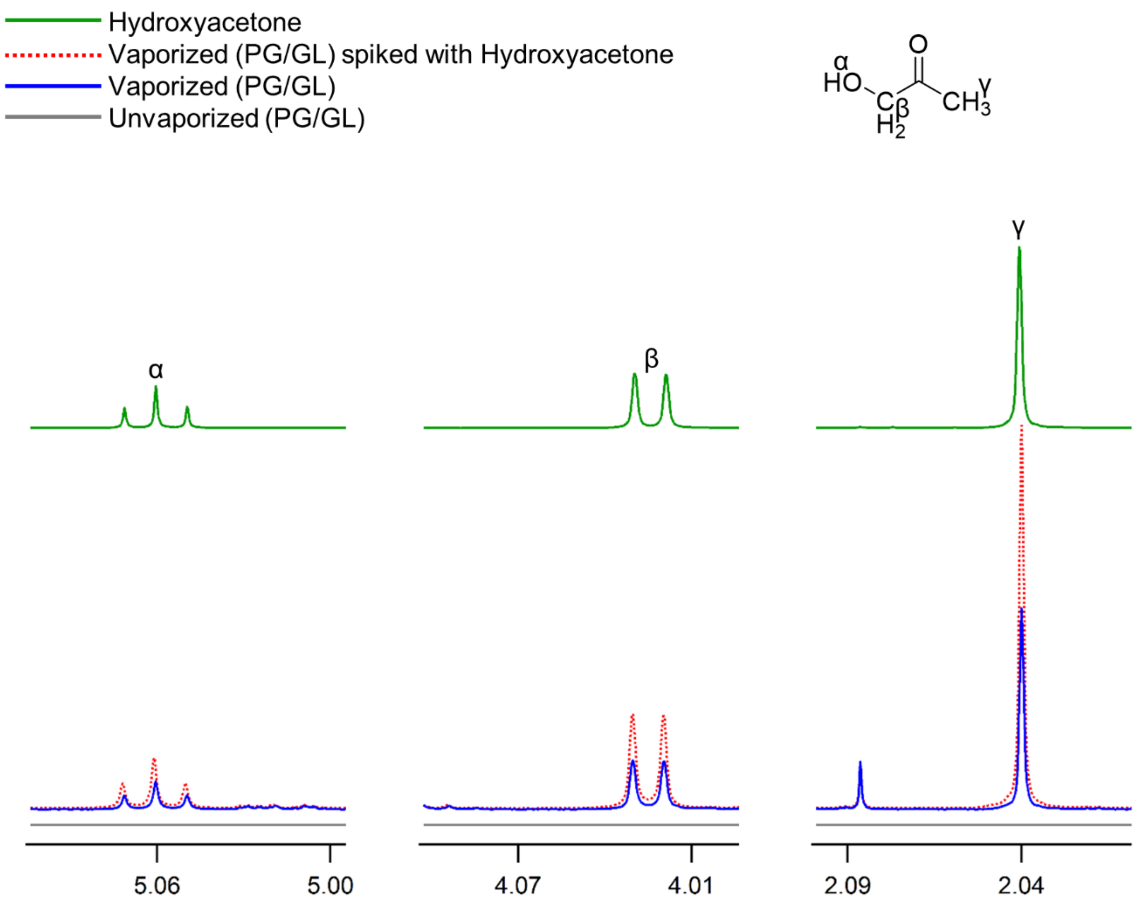

Figure S7 – ^{1}H NMR Assignment of Hydroxyacetone and Confirmation in Vaporized PG/GL.

Note that in the vaporized (PG/GL) spectrum, the small singlet peak corresponding to acetone (s, 2.08 ppm) does not change in intensity upon minute addition of hydroxyacetone to the vaporized (PG/GL) sample.

Acrolein

The simplest α,β-unsaturated aldehyde, acrolein is a double-dehydration product of glycerol which grows steadily in abundance as e-cigarette operating power is increased. The compound has distinct ^{1}H resonances that are easily recognized but unfortunately overlap with a number of compounds found in vaporized (PG/GL) samples. Having one proton in the α-position (the carbon bound to the carbonyl), the aldehyde resonance (d, 9.57 ppm) is split into a wide doublet. This α-proton (ddd, 6.34 ppm) is split into a complex resonance (a doublet-of-doublets-of-doublets) by the aldehyde and the two non-equivalent geminal protons on the adjacent β-carbon, which can themselves be assigned by their vicinal *cis* (dd, 6.62 ppm) and *trans* (dd, 6.48) coupling with the α-proton.

The aldehyde proton is sometimes partially occluded by a sharp singlet corresponding to monomeric formaldehyde. One of the geminal protons is usually un-occluded in the downfield region. Acrolein is often among the most abundant degradation species present in vaporized samples of (PG/GL) collected at high temperatures by using high power levels and long, slow puffs. The assigned ^{1}H NMR spectrum of acrolein is shown in Figure S8, along with confirmation of the compound in vaporized (PG/GL) by minute addition.

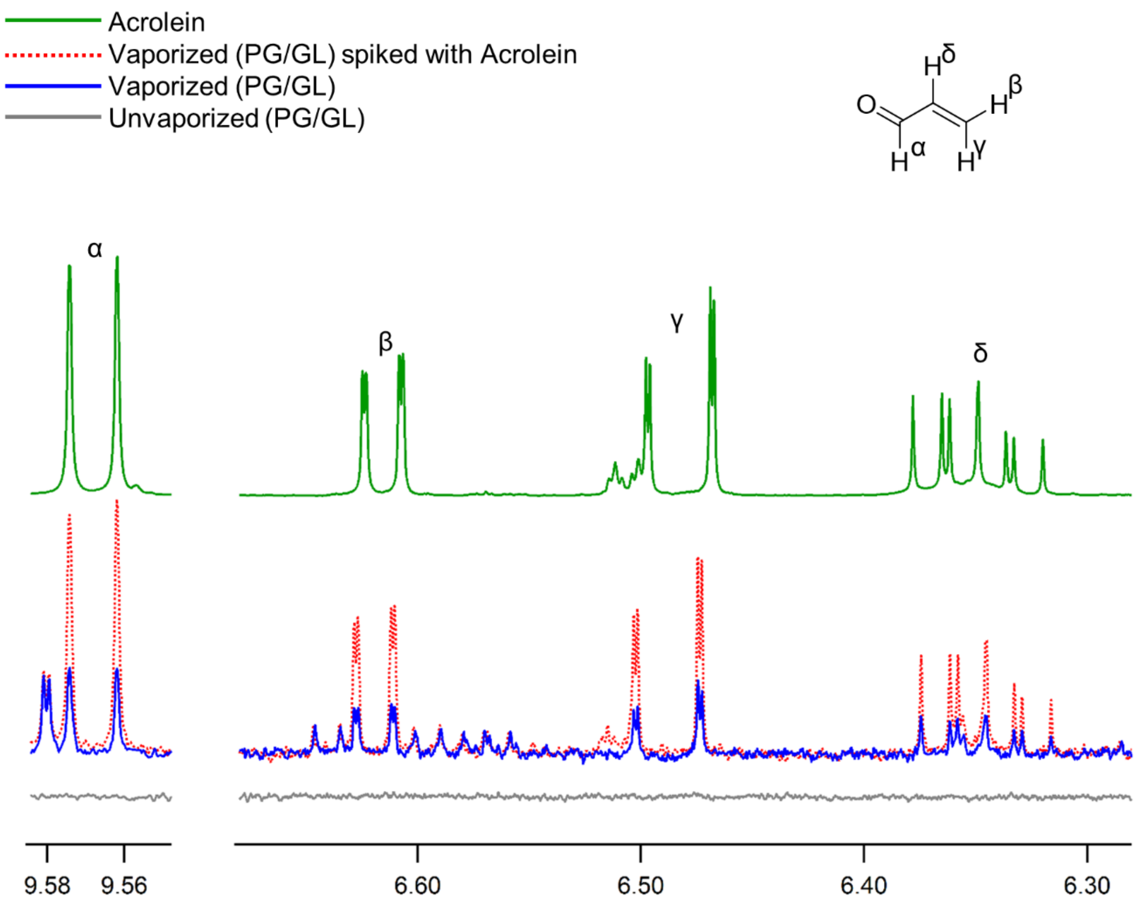

Figure S8 – ^{1}H NMR Assignment of Acrolein and Confirmation in Vaporized PG/GL.

Glycidol

Glycidol is a chiral oxirane compound with an unusual ^{1}H NMR spectrum. Two resonances corresponding to ring protons appear between the residual DMSO peak and the (PG/GL) methylene protons, the more upfield a doublet-of-doublets (dd, 2.66 ppm), and the more downfield a complex crown-shaped set of peaks (m, 2.98 ppm). At 400 MHz, these signals are unresolved from and occluded by the ^{13}C satellite peaks of the much larger resonances of DMSO and (PG/GL). A triplet corresponding to the hydroxyl proton (t, 4.84 ppm) is sometimes visible downfield of the (PG/GL) hydroxyls. The partially assigned ^{1}H NMR spectrum of glycidol is shown in Figure S9, along with confirmation of the compound in vaporized (PG/GL) by minute addition. Glycidol is a known dehydration product of glycerol. This compound never grows to a very high abundance, possibly due to its inherent reactivity.

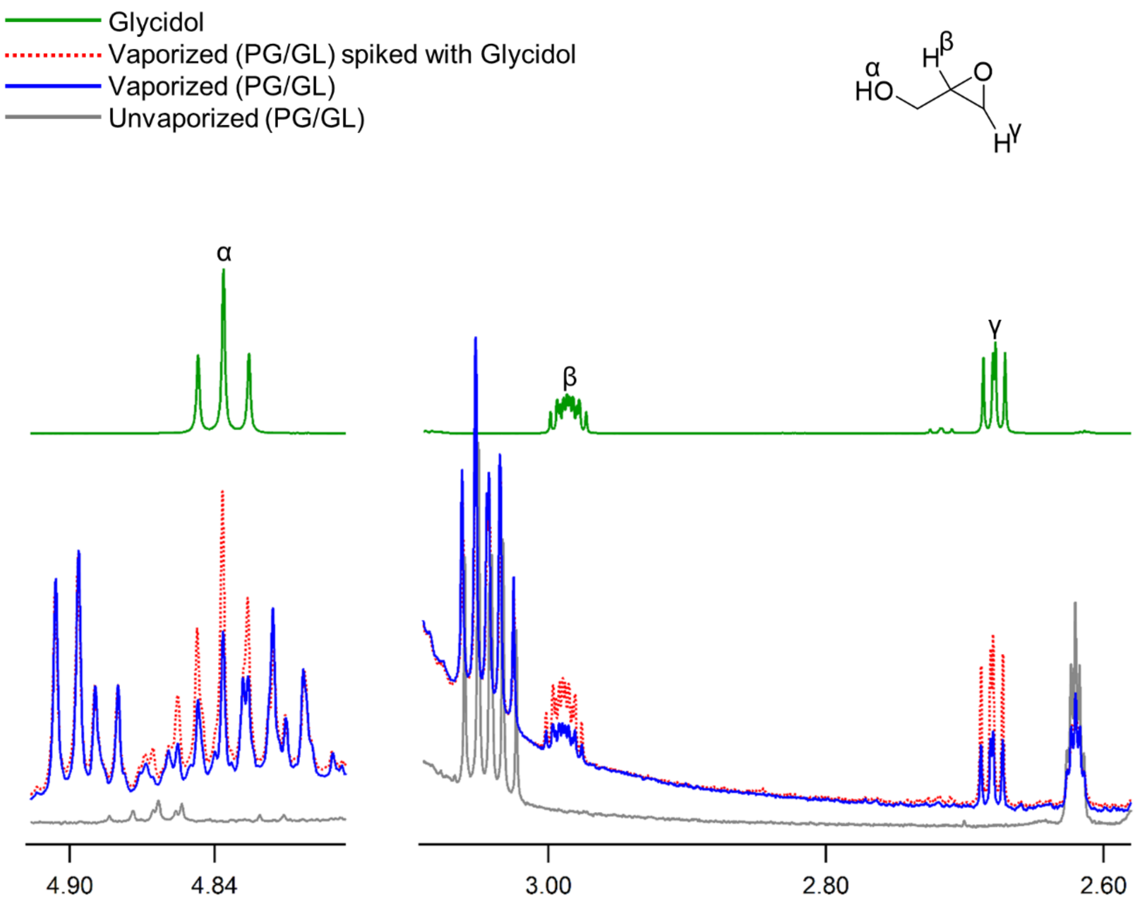

Figure S9 – ^{1}H NMR Assignment of Glycidol and Confirmation in Vaporized PG/GL.

Acetaldehyde

Acetaldehyde is easy to recognize by ^{1}H NMR spectroscopy – the aldehyde resonance is split into an unmistakable quartet (q, 9.65 ppm) by the three adjacent acetyl protons, while the acetyl resonance (d, 2.12 ppm) is split by only the single aldehyde proton into a doublet. The assigned ^{1}H NMR spectrum of acetaldehyde is shown in Figure S10, along with confirmation of the compound in vaporized (PG/GL) by minute addition. These resonances have a strong correlation by ^{1}H-^{1}H COSY, and their attachments may be observed by ^{1}H-^{13}C HSQC and HMBC experiments. This two-carbon compound is a secondary degradation product that grows to very high abundances in samples of (PG/GL) vaporized at high power levels.

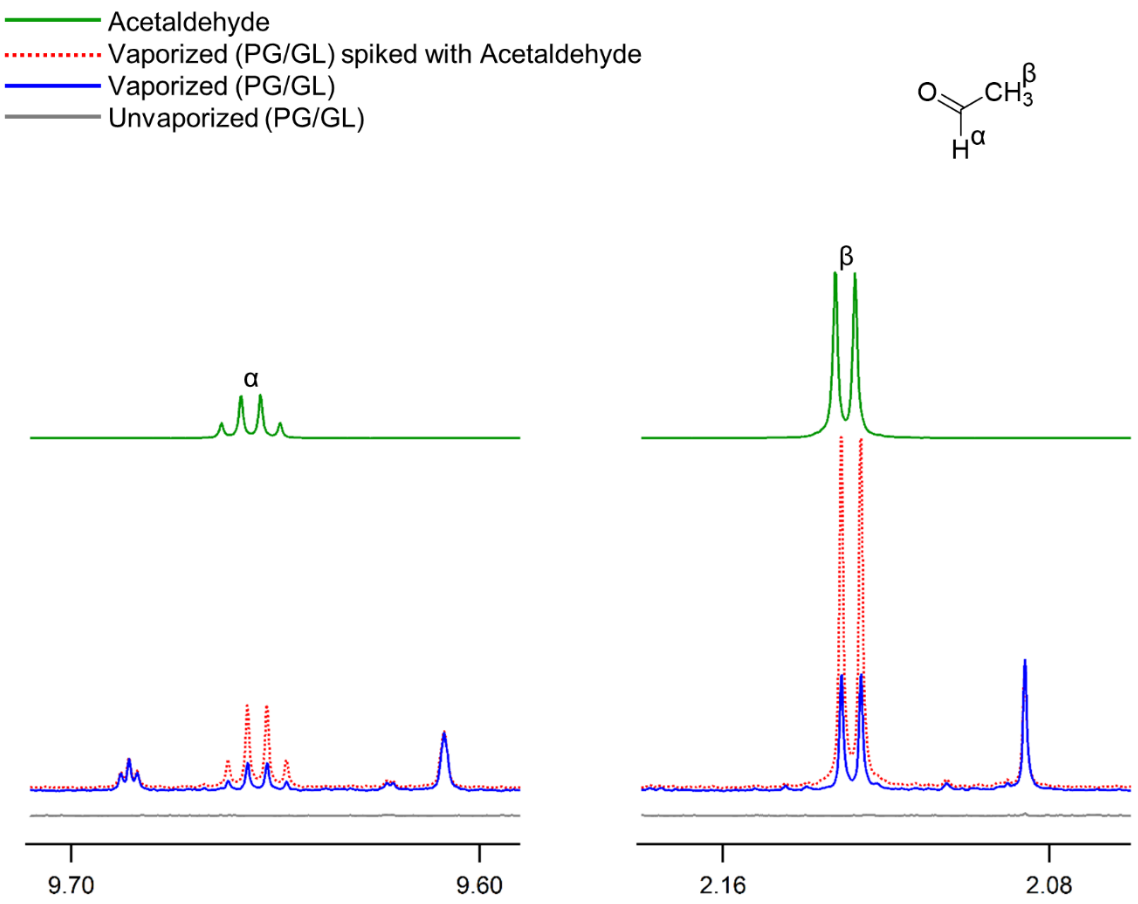

Figure S10 – ¹H NMR Assignment of Acetaldehyde and Confirmation in Vaporized PG/GL.

Formaldehyde

Formaldehyde is a volatile and reactive molecule that is not usually encountered in its monomeric form. The sample collection technique described above does allow for the observation of a sharp singlet peak in the aldehyde region that corresponds to the minor peak present in the ^{1}H spectrum of formalin solution diluted in DMSO-D_6. In a ^{1}H spectrum collected from a sample of vaporized (PG/GL), this sharp aldehyde resonance overlaps partially with the aldehyde resonance of acrolein before diminishing in intensity over the course of several hours and ultimately disappearing entirely from view. This disappearance is coincident with the growth of peaks corresponding to hemiformals of glycerol and propylene glycol, indicating that the carbonyl formaldehyde present has formed hemiacetals with the propanols it encountered while in solution.

Dihydroxyacetone

Dihydroxyacetone is a symmetrical molecule with an extremely simple ^{1}H NMR spectrum, making it difficult to assign certainly without confirmation by spiking. The signals are very similar to those of hydroxyacetone, but for the absence of an acetyl singlet. One can use ^{1}H-^{13}C Heteronuclear Multiple Bond Correlation (HMBC) experiments to correlate the methylene protons (d, 4.16 ppm) with a carbon in the ketone region of the ^{13}C spectrum. The hydroxyl triplet (t, 5.02 ppm) sits immediately upfield from the nearly identical hydroxyl triplet of hydroxyacetone and is sometimes occluded by ^{1}H resonances from allyl alcohol. The pure form of dihydroxyacetone is often encountered as a dimer and must be dissociated with elevated temperature in dilute solution before it can be used to confirm the presence of the monomer

by minute addition. The assigned ^{1}H NMR spectrum of dihydroxyacetone is shown in Figure S11, along with confirmation of the compound in vaporized (PG/GL) by minute addition.

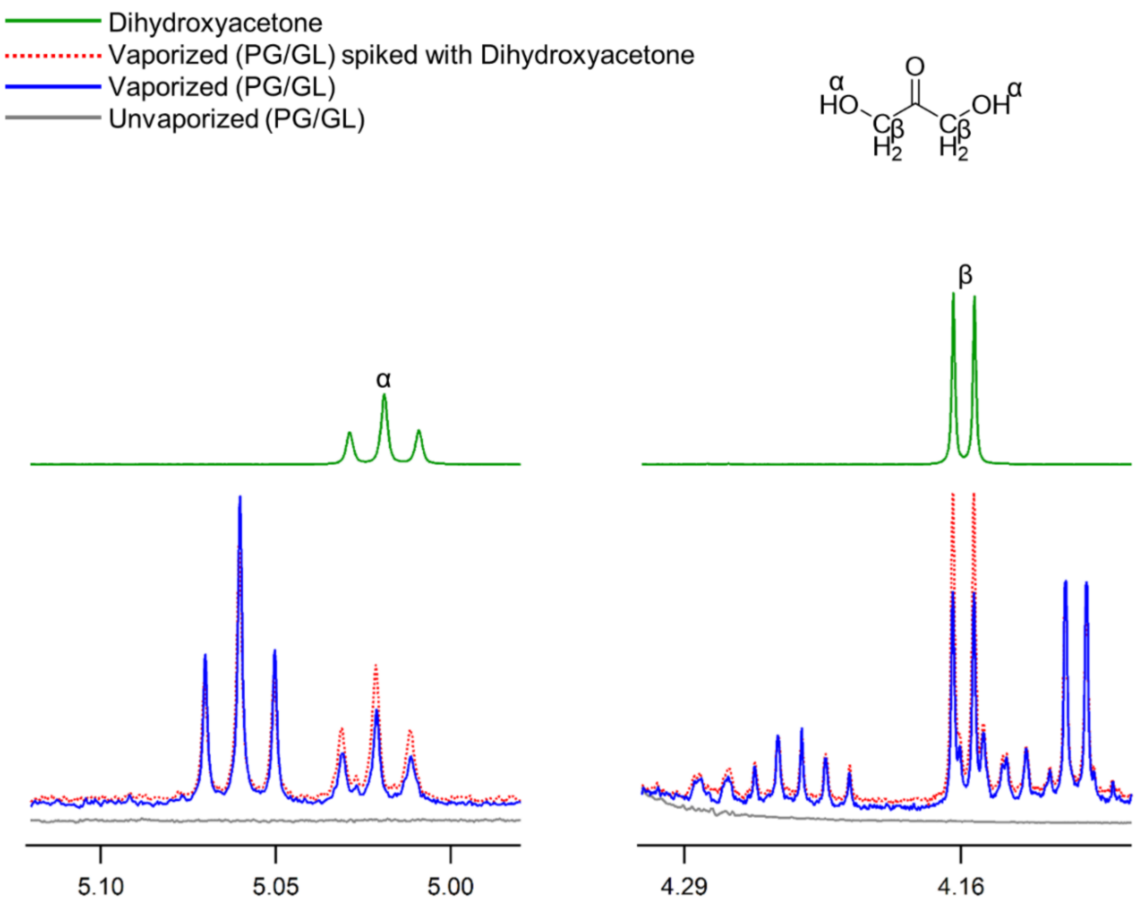

Figure S11 – ^{1}H NMR Assignment of Dihydroxyacetone and Confirmation in Vaporized PG/GL.

Glyceraldehyde

Glyceraldehyde is an α,β-dihydroxyaldehyde that exists as a solid dimer that can be dissociated with elevated temperature and will persist as a monomer in solution if sufficiently dilute. The aldehyde resonance of glyceraldehyde (d, 9.62 ppm) is a very tightly coupled doublet – a trait that I have found typical of α-hydroxyaldehyhdes. Perhaps unsurprisingly, most of the non-aldehyde protons present in glyceraldehyde resonate at frequencies very similar to (PG/GL) and are not well resolved. Glyceraldehyde is never among the most abundant decomposition products of vaporized glycerol and is possibly susceptible to dissociation by a retro-aldol mechanism. The partially assigned ^{1}H NMR spectrum of glyceraldehyde is shown in Figure S12, along with confirmation of the compound in vaporized (PG/GL) by minute addition.

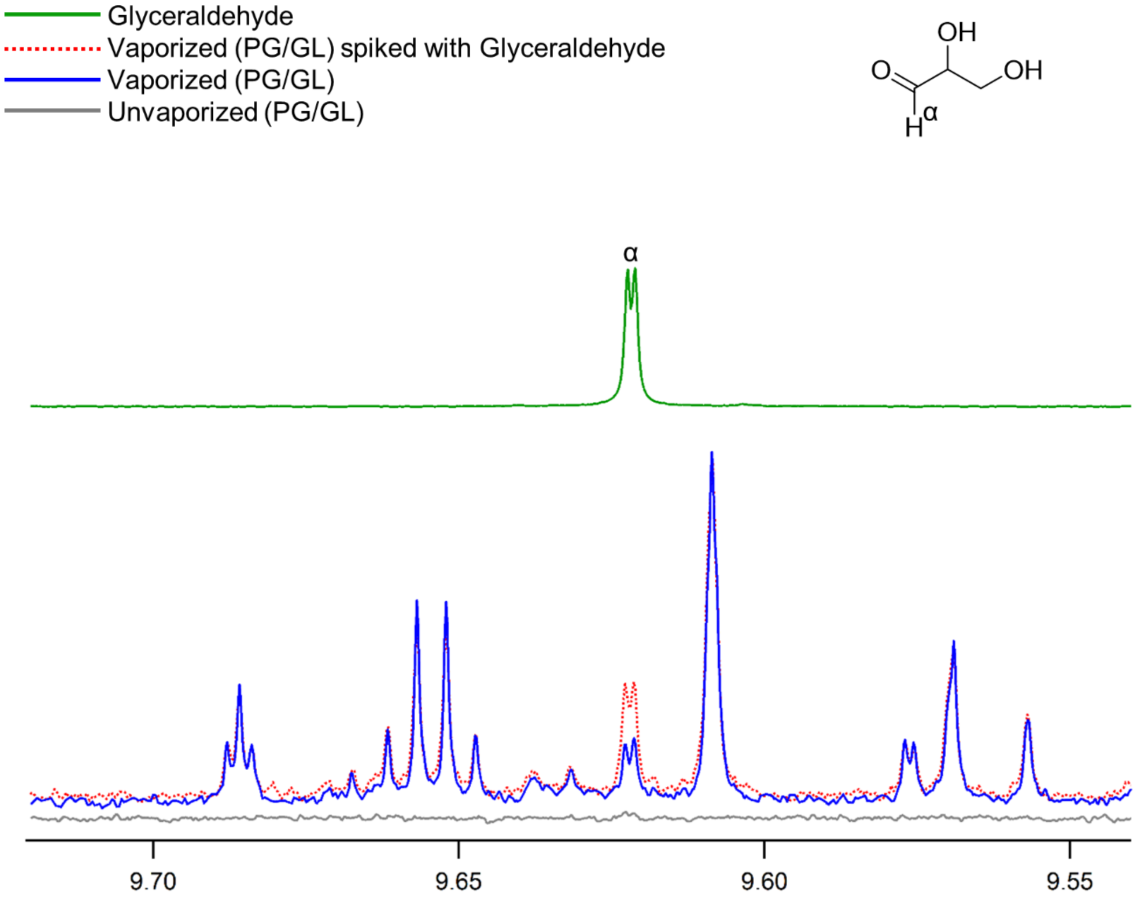

Figure S12 – ^{1}H NMR Assignment of Glyceraldehyde and Confirmation in Vaporized PG/GL.

Glycolaldehyde

Glycolaldehyde is the simplest α-hydroxyaldehyde and has a very simple ^{1}H NMR spectrum. One might properly refer to glycolaldehyde as the simplest sugar molecule. This compound is often present in samples of (PG/GL) that have been vaporized under low-temperature conditions – it is the earliest of the two-carbon species to arise, sometimes present in samples lacking any detectable acetaldehyde or acetic acid. The structure of glycolaldehyde is shown in Figure S13.

Like glyceraldehyde and lactaldehyde, which are also α-hydroxyaldehydes, the aldehyde resonance of glycolaldehyde is very tightly coupled to the adjacent protons. The aldehyde resonance (t, 9.61 ppm) appears to be a singlet to a first approximation, but has triplet character upon close inspection. The methylene protons (d, 4.10 ppm) are split into a doublet by the hydroxyl proton (t, 5.30 ppm).

Like dihydroxyacetone and glyceraldehyde, the pure form of this compound is usually obtained as a solid dimer that can be dissociated with heat. The fact that this compound is an intermediate between formaldehyde and glyceraldehyde in the formose reaction makes retro-aldol dissociation – similar to the dissociation of hydroxyacetone into acetaldehyde and formaldehyde described by Nef – a possibility for the mechanism of glycolaldehyde formation. The assigned ^{1}H NMR spectrum of glycolaldehyde is shown in Figure S13, along with confirmation of the compound in vaporized (PG/GL) by minute addition.

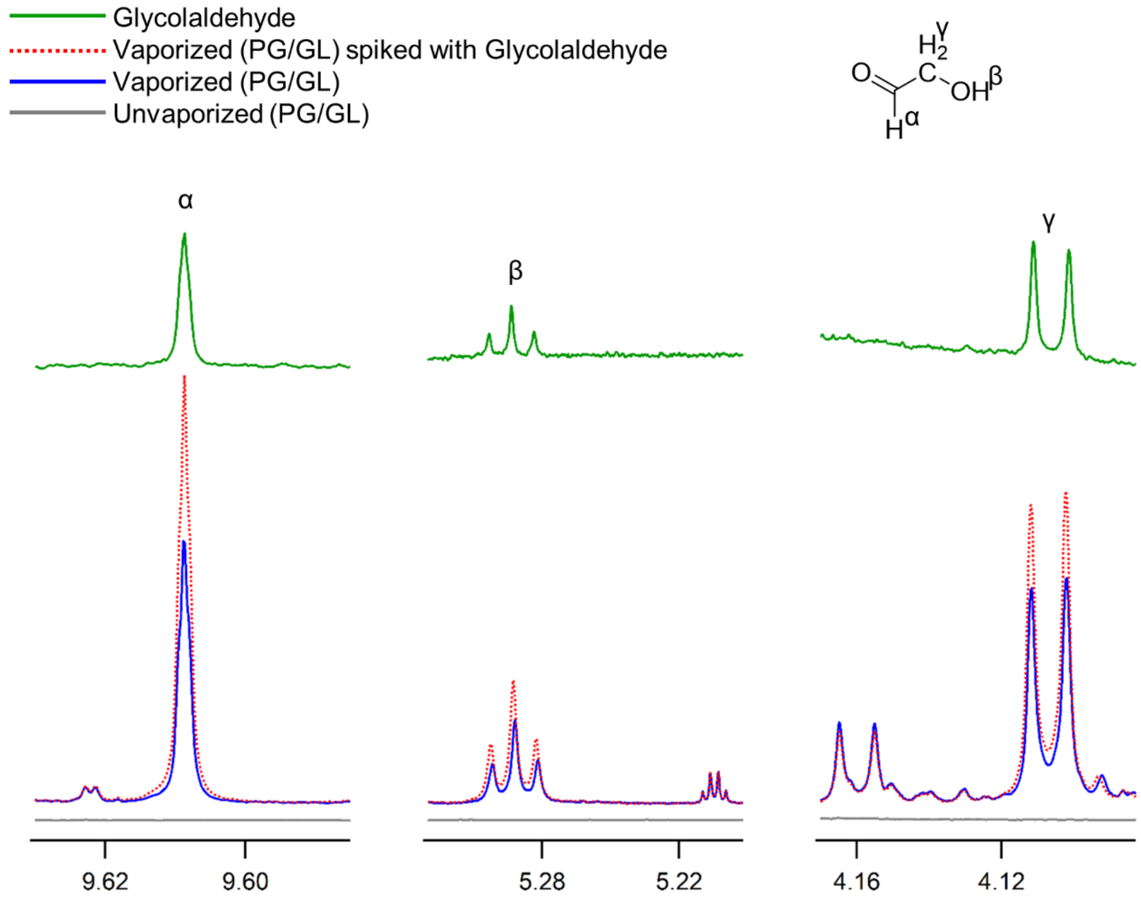

Figure S13 – ^{1}H NMR Assignment of Glycolaldehyde and Confirmation in Vaporized PG/GL.

To determine whether glycolaldehyde might result from glyceraldehyde decomposition, a sample of glyceraldehyde was dissolved to approximately 10% by mole in propylene glycol. Glycolaldehyde does not form as a result of pure propylene glycol decomposition, but was observed as a product in this glyceraldehyde-doped sample, making glyceraldehyde a possible intermediate between glycerol and glycolaldehyde.

Allyl alcohol

Allyl alcohol is both a decomposition product of glycerol *and* a dehydration product of propylene glycol. The geminal protons (*trans*: dq, 5.19 ppm; *cis*: dq, 5.02 ppm) of the alkene appear distinctly as doublets-of-quartets, being split by one another, the vicinal proton, and the nearby methylene protons. The lone proton (tt, 5.91 ppm) on the middle carbon appears as a triplet-of-triplets, being split by the adjacent methylene protons and by the two terminal alkene protons. The methylene protons (m, 3.93 ppm) are split by many protons and appear as a complex crown-shaped resonance. The hydroxyl proton (t, 4.71 ppm) is a sharp triplet, being split by the two adjacent methylene protons. The assigned ^{1}H NMR spectrum of allyl alcohol is shown in Figure S14, along with confirmation of the compound in vaporized (PG/GL) by minute addition.

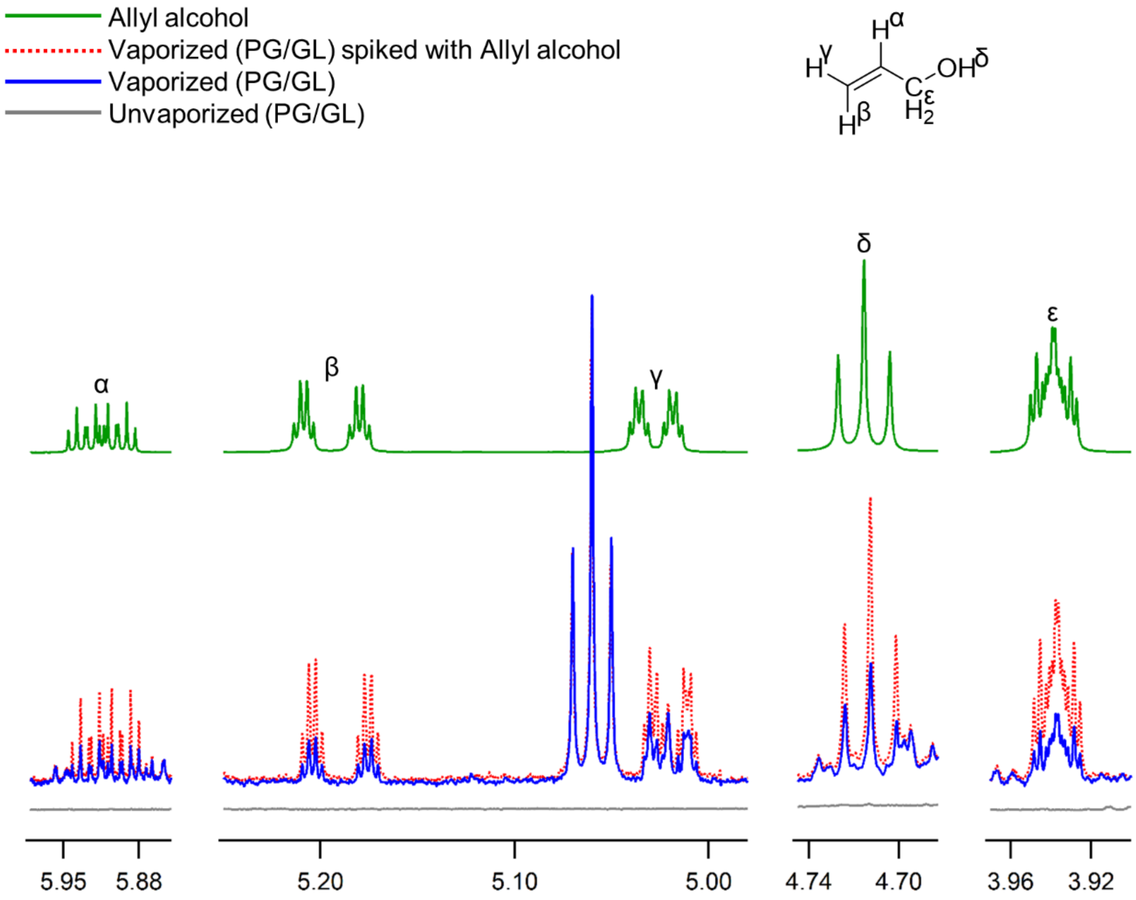

Figure S14 – ^{1}H NMR Assignment of Allyl Alcohol and Confirmation in Vaporized PG/GL.

The mechanism of allyl alcohol formation from glycerol is not straightforward; neither a simple dehydration nor a simple oxidation, glycerol must lose H_2O_2 in order to become allyl alcohol. This transformation may occur in several steps and could be mediated by reaction with formic acid, as has been reported previously.

Acetic Acid
Acetic acid is a decomposition product of both glycerol and propylene glycol. The ^{1}H NMR spectrum of acetic acid contains a sharp singlet (s, 1.91 ppm) in the acetyl region and a broad singlet in the carboxylic acid region – the acid peak is not particularly useful, being broad due to rapid exchange and erratic in chemical shift. The partially assigned ^{1}H NMR spectrum of acetic acid is shown in Figure S15, along with confirmation of the compound in vaporized (PG/GL) by minute addition.

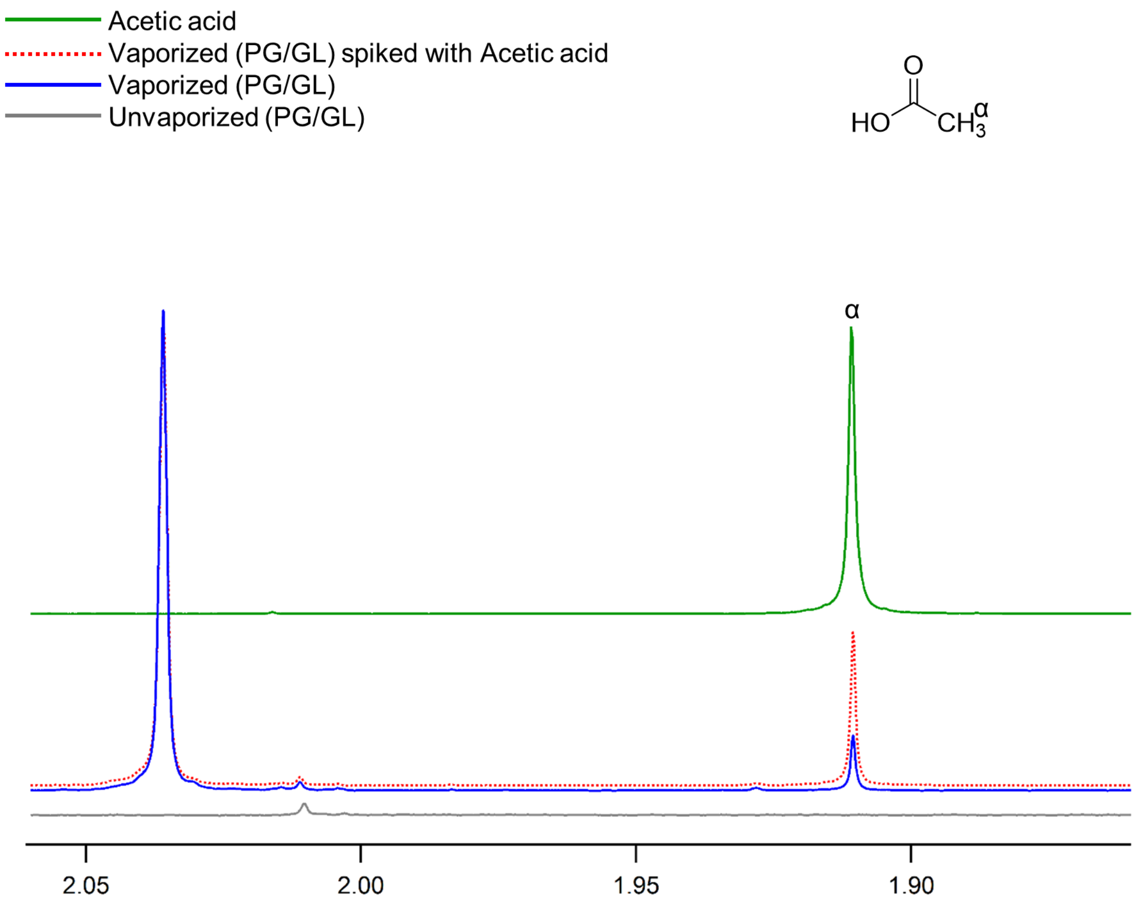

Figure S15 – ^{1}H NMR Assignment of Acetic Acid and Confirmation in Vaporized PG/GL.

Glycerol itself contains no methyl carbon – only methylenes and a methyne – so acetic acid must be a secondary decomposition product of hydroxyacetone or some other compound that has already formed a methyl carbon by dehydration and rearrangement, or it must result from a multi-step process where such a rearrangement occurs.

Formic Acid

Formic acid has a sharp singlet (s, 8.14 ppm) corresponding to the aldehyde proton. The partially assigned ^{1}H NMR spectrum of formic acid is shown in Figure S16, along with confirmation of the compound in vaporized (PG/GL) by minute addition.

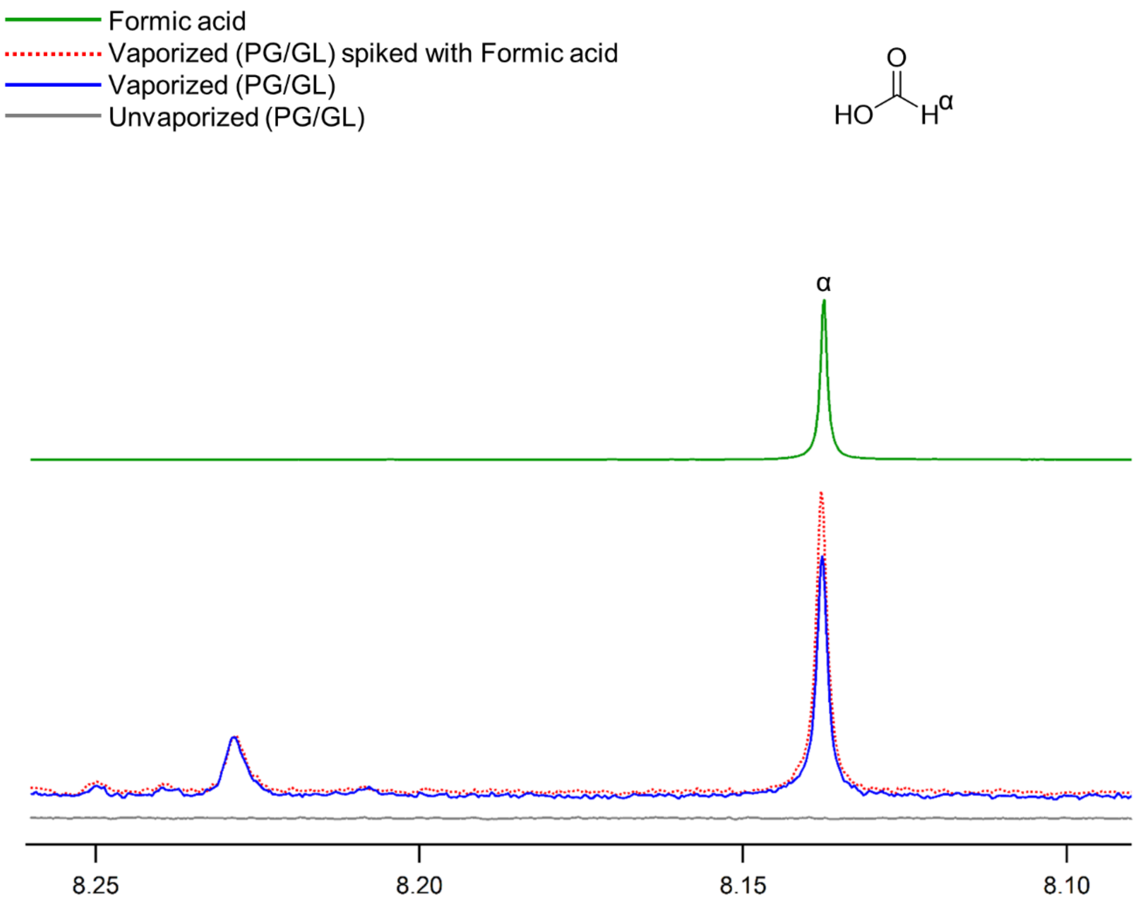

Figure S16 – ^{1}H NMR Assignment of Formic Acid and Confirmation in Vaporized PG/GL.

Formic acid may result from elimination of a carbon-carbon bond by a glycerol oxy-alkyl radical, separating from the other two carbons, which may rearrange into a stable product such as glycolaldehyde or acetaldehyde, or may further decompose into volatile single-carbon products such as formaldehyde, carbon monoxide, or carbon dioxide.

Propanal

Propanal has three ^{1}H resonances that are visible in samples of vaporized (PG/GL) corresponding to the aldehyde (t, 9.68 ppm), the α-methylene protons (dq, 2.45 ppm), and the methyl protons (t, 0.96 ppm) – the methyl triplet is sometimes occluded by the overwhelmingly-large PG methyl triplet of similar chemical shift. Unless samples are very concentrated, the doublet-of-quartets corresponding to the α-methylene protons is not fully resolved from the DMSO-D_5H peak at 2.50 ppm. The partially assigned ^{1}H NMR spectrum of propanal is shown in Figure S17, along with confirmation of the compound in vaporized (PG/GL) by minute addition.

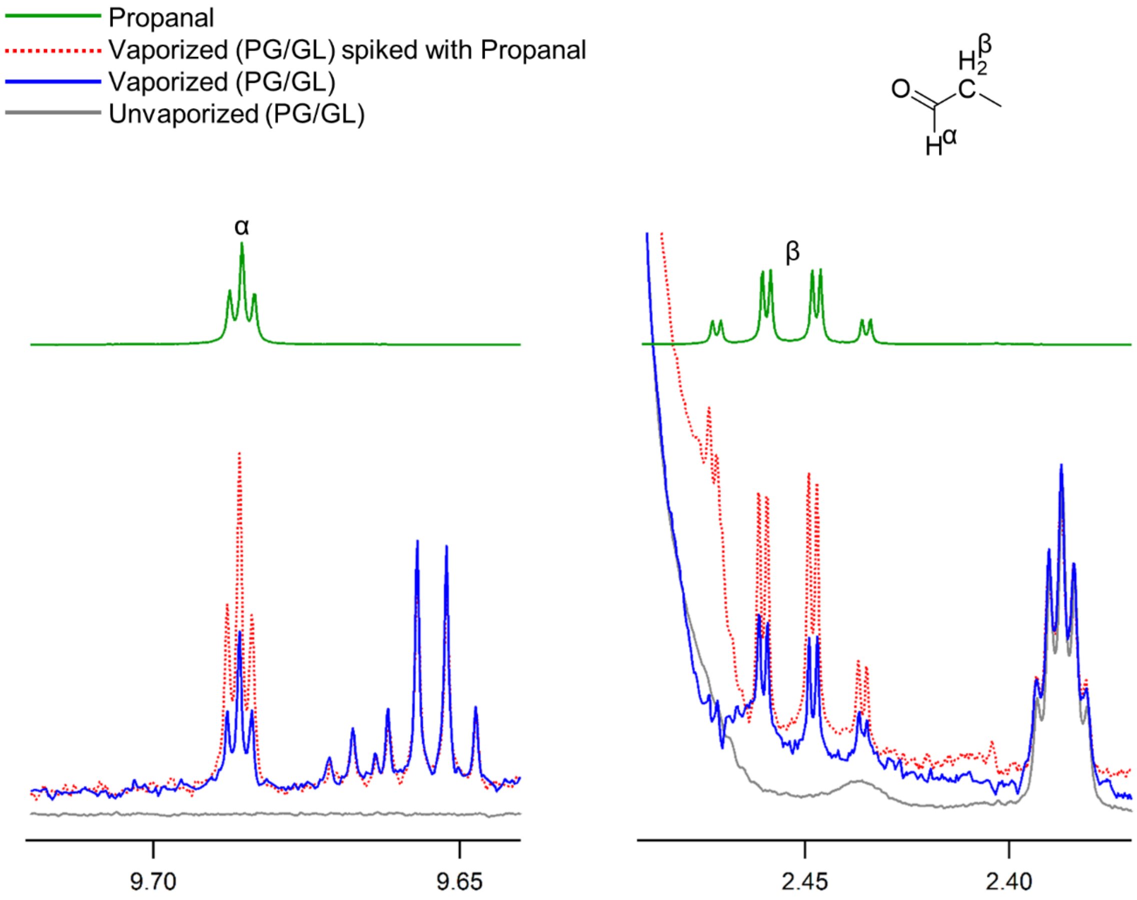

Figure S17 – ¹H NMR Assignment of Propanal and Confirmation in Vaporized PG/GL.

E,Z-prop-1-ene-1-ol

These vinyl alcohols are enols – unstable intermediates that form during the dehydration of propylene glycol and tautomerize to form propanal. The conditions under which samples are collected and analyzed in this investigation leads to the rapid dilution of vaporized (PG/GL) and their degradation products, allowing reactive species such as these prop-1-ene-1-ols to persist for hours or days before rearranging into the more stable carbonyl compound. This rearrangement can be observed by ^{1}H NMR experiments and can be accelerated by heating if the probe being used is capable of controlling and varying temperature. The separation and detection of these vinyl alcohols by gas chromatography may be difficult due to their tautomerization to propanal unless care is taken to keep the inlet and oven temperatures low. The ^{1}H NMR assignments of *E,Z*-prop-1-ene-1-ol and evidence of their tautomerization to propanal are summarized in Figure S18. In this figure, unvaporized propylene glycol is plotted in grey, indicating that all the peaks shown are degradation products; in blue is plotted a spectrum of vaporized propylene glycol collected immediately after sample collection, and in red is plotted a spectrum of that same sample collected after 24 hours at 25 °C. Resonances corresponding to the enols are plotted on top and diminish over time, while those corresponding to propanal (for which ^{1}H NMR assignments are given in Figure 3.29) are plotted on bottom and grow over time.

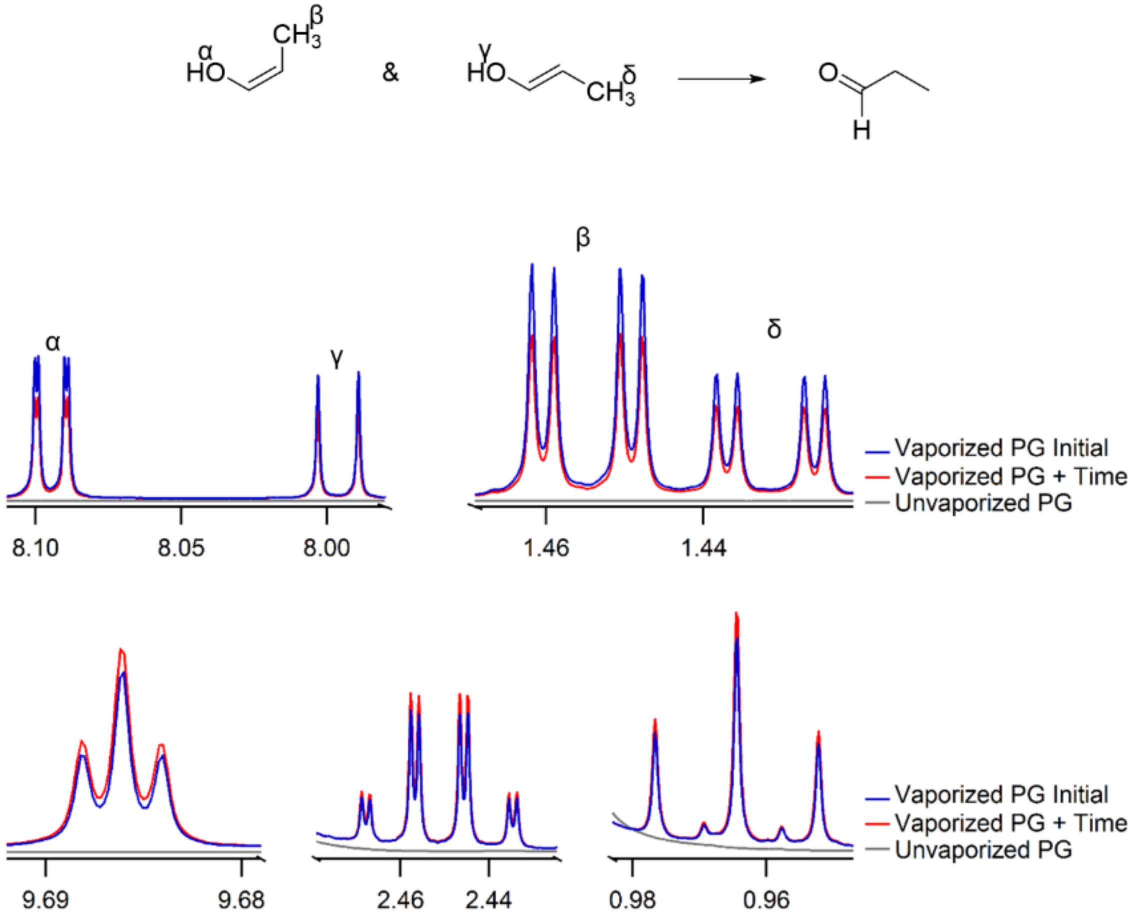

Figure S18 – Tautomerization of *E,Z*-prop-1-ene-1-ol to Propanal in Vaporized PG/GL.

Acetone

Acetone has only a single ^1H resonance (s, 2.09 ppm) making it particularly unobvious in a spectrum of vaporized (PG/GL) containing dozens of peaks. This resonance can be correlated to an acetyl carbon and a ketone using HSQC and HMBC experiments; the only way to confirm that this resonance is from acetone specifically is to spike the sample with a minute addition of the pure compound. The assigned ^1H NMR spectrum of acetone is shown in Figure S19, along with confirmation of the compound in vaporized (PG/GL) by minute addition.

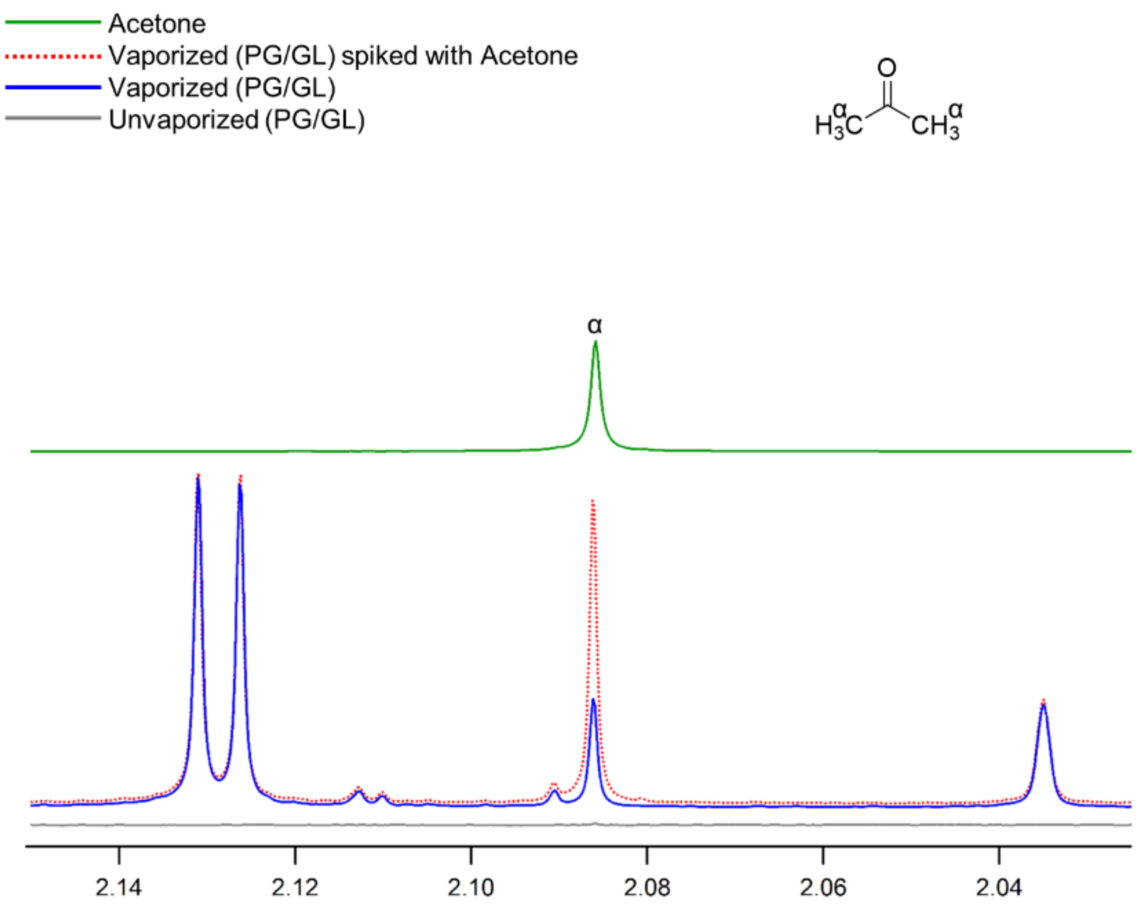

Figure S19 – ^1H NMR Assignment of Acetone and Confirmation in Vaporized PG/GL.

Lactaldehyde

Lactaldehyde is an α-hydroxyaldehyde – like glyceraldehyde, the aldehyde resonance is a very tightly coupled doublet. Perhaps unsurprisingly, most of the non-aldehyde protons present in lactaldehyde resonate at frequencies very similar to (PG/GL) and are not well resolved. The partially assigned ^{1}H NMR spectrum of lactaldehyde is shown in Figure S20, along with confirmation of the compound in vaporized (PG/GL) by minute addition.

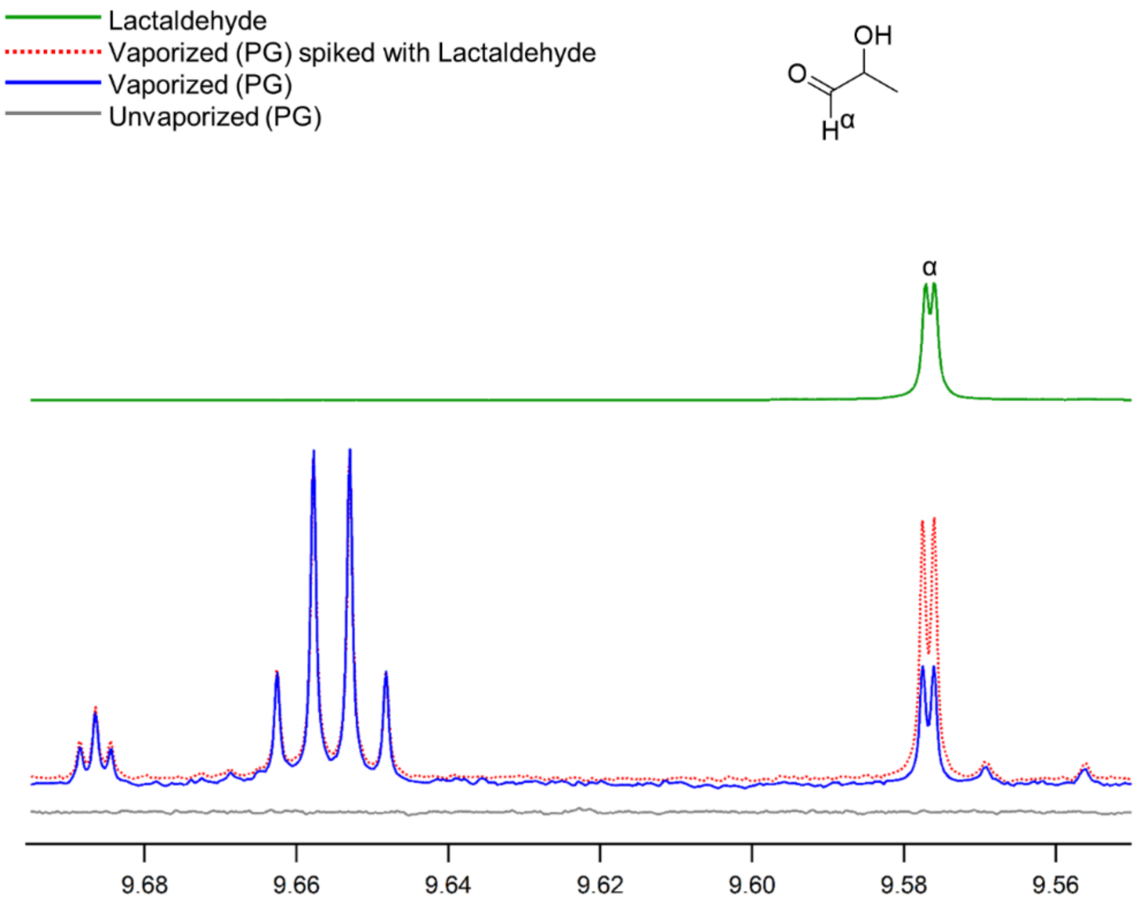

Figure S20 – ^{1}H NMR Assignment of Lactaldehyde and Confirmation in Vaporized PG/GL.

Formaldehyde releasing agents (FRAs): details

These assignments were made by forming the species in pure PG or GLY. The primary and secondary labeling was distinguished for the GLY case by forming the corresponding compound from the 1-methoxy-2-propanol compound (from TCI America), verifying that the most readily formed species is the primary label is for the primary addition of the hemiacetal. The primary formation of the hemiacetal of the corresponding GLY compound has a very similar chemical shift, as well as high abundance.

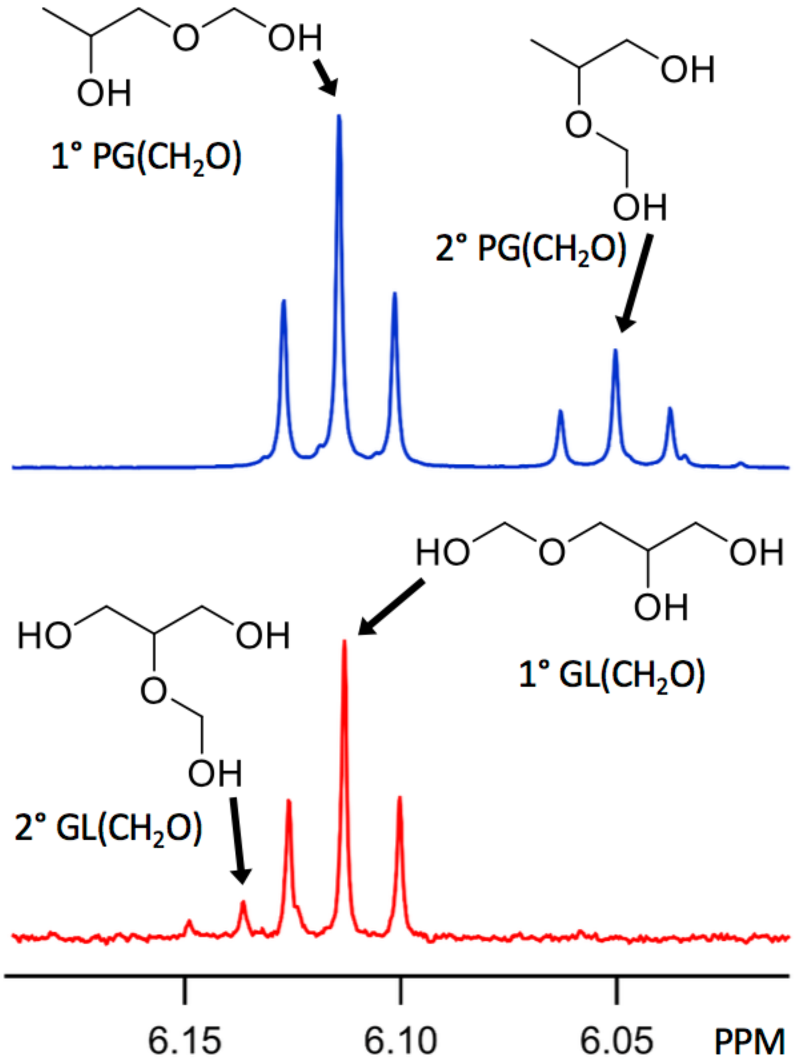

Figure S21 – ^{1}H NMR Assignments of various FRA hemiacetals of formaldehyde with PG and GLY (GL) in vaporized PG/GL.

The Effect of Oxygen on Solvent Decomposition

When measures are taken to limit the amount of oxygen present during collection of vaporized (PG/GL) samples, an obvious decrease in the intensity of many ^{1}H NMR peaks corresponding to decomposition products can be observed. Samples of vaporized (PG/GL) were collected in a sealed glove-bag that had been flushed with compressed nitrogen until a portable oxygen detector (BW Technologies GAXT-X-DL-2) indicated that O_2 levels were < 0.1% of the atmosphere inside the bag. Anaerobic samples were collected in between aerobic samples as indicated in Table S4, which details the order the samples were collected in, the concentration of oxygen present in the glove-bag during collection of each sample, and the mass of each sample collected in the glass vial. The consumption of (PG/GL) was not possible to determine during this experiment because the clearomizer could not be removed from the glove-bag after each puff to determine its mass. The average of the six normalized ^{1}H NMR spectra from each condition are plotted in Figure S21.

Table S4 – Conditions and Consumption of Aerobic/Anaerobic Samples.

Sample #	$[O_2]$ (%vol)	Mass Collected (mg)
1	20.9	30
2	20.9	18
3	20.9	25
4	<0.1	26
5	<0.1	34
6	<0.1	34
7	<0.1	42
8	<0.1	37
9	<0.1	31
10	20.9	25
11	20.9	14
12	20.9	23

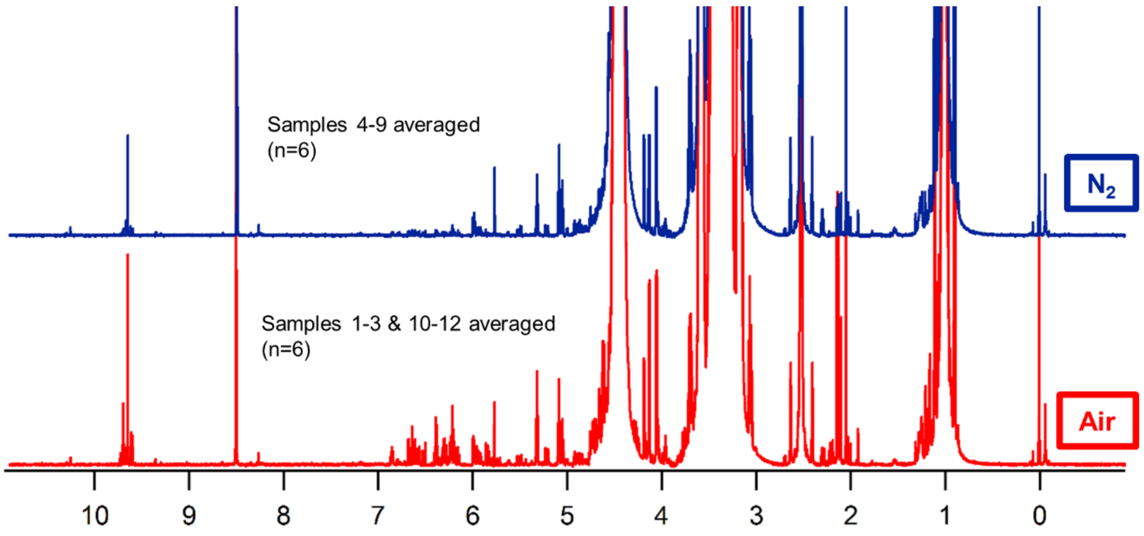

Figure S21 – Averaged ^{1}H Spectra (PG/GL) Vaporized Under Anaerobic and Aerobic Conditions.

The intensities of many peaks in the spectrum of the sample collected under nitrogen flow are significantly reduced when compared with samples collected under air flow, despite the fact that the samples collected under nitrogen are generally heavier than those collected under air. Some of the peaks associated with decomposition products in the anaerobic spectra, including glycolaldehyde and hydroxyacetone, appear to be less influenced by the absence of oxygen than others. These two species were observed to be the most abundant decomposition products in the anaerobic samples detailed in Table 4– in fact, they were the only two decomposition products which were present in all twelve samples collected during both conditions of this experiment. Thus, these species were quantified in all samples by relative integration against 1.0 mM 1,2,4,5-tetrachloro-3-nitrobenzene. The results of this quantification are presented in Figures S22 and S23, which show each sample treated independently and averaged together by condition.

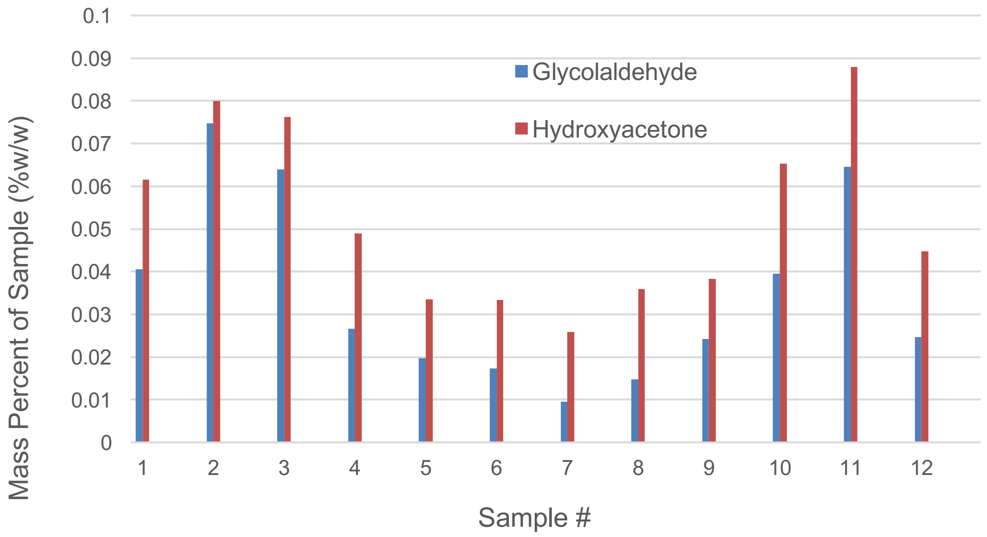

Figure S22 – Quantification of Hydroxyacetone and Glycolaldehyde in Anaerobic and Aerobic Samples.

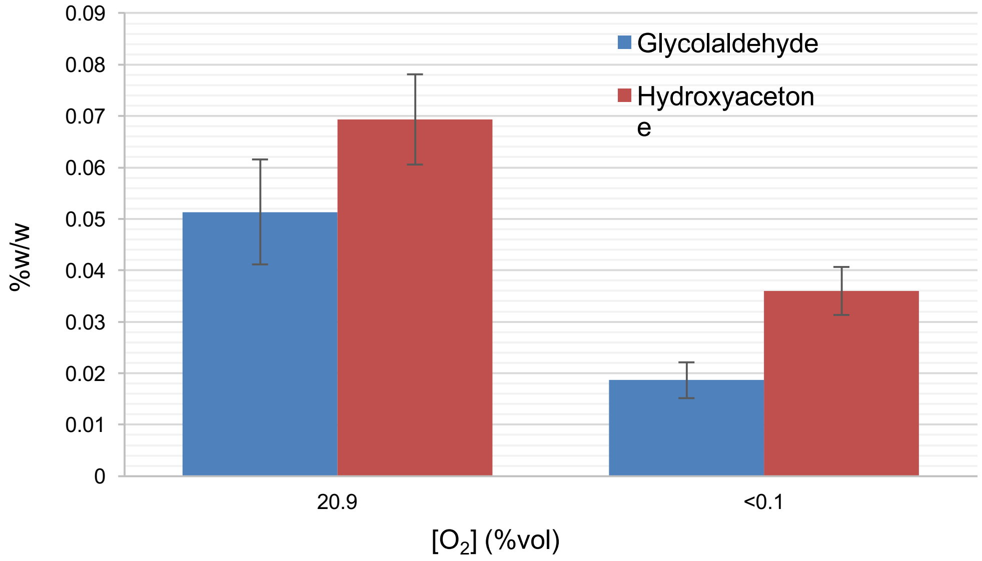

Figure S23 – Averaged Values of Hydroxyacetone and Glycolaldehyde in Anaerobic and Aerobic Samples.

Considering that glycolaldehyde and hydroxyacetone are the decomposition products that remained most abundant in the anaerobic samples, these results indicate that removal of oxygen from the system has the general effect of decreasing the amount of decomposition in vaporized samples of (PG/GL). Similar treatment of other decomposition products such as acrolein, acetaldehyde, or allyl alcohol, which were not present at detectable levels in several of the anaerobic samples, would demonstrate a more striking but less generalizable contrast.
